# Multidisciplinary consensus on prevention, screening and monitoring of clozapine-associated myocarditis and clozapine rechallenge after myocarditis

**DOI:** 10.1192/bjp.2025.89

**Published:** 2026-04

**Authors:** Elias Wagner, Nicole Korman, Marco Solmi, Matin Mortazavi, Zahra Aminifarsani, Douglas Dubrovin Leão, Matthew K. Burrage, Dan Siskind, Laura McMahon, Oliver D. Howes, Christoph U. Correll, CAM Expert Group, Alkomiet Hasan

**Affiliations:** Evidence-based Psychiatry and Psychotherapy, Faculty of Medicine, University of Augsburg, Augsburg, Germany; Department of Psychiatry, Psychotherapy and Psychosomatics, Medical Faculty, University of Augsburg, BKH Augsburg, Augsburg, Germany; Queensland Centre for Mental Health Research, Brisbane, Australia; Metro South Addiction and Mental Health Service, Metro South Health, Brisbane, Australia; University of Queensland School of Clinical Medicine, Brisbane, Australia; Department of Psychiatry and Ottawa Hospital Research Institute Clinical Epidemiology Program, University of Ottawa, Ottawa, Canada; Regional Centre for the Treatment of Eating Disorders and On Track: The Champlain First Episode Psychosis Program, Department of Mental Health, The Ottawa Hospital, Ottawa, Canada; Department of Child and Adolescent Psychiatry, Charité Universitätsmedizin, Berlin, Germany; Faculty of Medicine, University of Queensland, Brisbane, Australia; Department of Cardiology, Ipswich Hospital, Ipswich, Australia; Queensland Brain Institute, University of Queensland, Brisbane, Australia; Mental Health Service, Darling Downs Health District, Toowoomba, Australia; Institute of Psychiatry, Psychology and Neuroscience, King’s College London, London, UK; Department of Psychiatry, Zucker Hillside Hospital, Glen Oaks, NY, USA; Departments of Psychiatry and Molecular Medicine, Donald and Barbara Zucker School of Medicine at Hofstra/Northwell, Hempstead, NY, USA; DZPG (German Center for Mental Health), Partner Site München/Augsburg, Augsburg, Germany

**Keywords:** Clozapine, myocarditis, cardiotoxicity, schizophrenia, treatment resistance

## Abstract

**Background:**

Clozapine is the antipsychotic of choice for people with treatment-resistant schizophrenia (TRS) but is associated with the uncommon but potentially life-threatening adverse effect of myocarditis. However, there are no criteria for diagnosing clozapine-associated myocarditis (CAM) or global guidelines on detection and risk reduction, or for restarting clozapine after CAM.

**Aims:**

To develop criteria for CAM and algorithms for clozapine initiation and clozapine rechallenge after CAM in a multiprofessional consensus process.

**Method:**

We conducted a systematic literature search for cases of clozapine rechallenge following CAM using the PubMed, EMBASE, CINAHL and PsycINFO databases, followed by a multidisciplinary international two-step Delphi consensus process in July and October 2024. The Delphi panel comprised psychiatrists, cardiologists, pharmacists, psychopharmacologists and nurses with expertise on clozapine or myocarditis.

**Results:**

Ninety-three clinicians and academics with experience in prescribing clozapine from six continents participated in the Delphi process. A consensus was reached on a definition of CAM according to modified clinical criteria from the European Society of Cardiology for myocarditis associated with immune checkpoint inhibitors. Titration schemes slower than those given in the Summary of Product Characteristics for clozapine were recommended to minimise CAM risk. Minimum and enhanced requirements for screening and monitoring were developed to account for global perspectives and limited resources in certain healthcare systems, and an approach to clozapine rechallenge was elaborated.

**Conclusions:**

This multidisciplinary project represents the first guidance for CAM and will inform clinicians, other caregivers and patients, as well as facilitating the development of national guidelines on CAM prevention, screening and monitoring and rechallenge after an index episode of myocarditis in individuals taking clozapine.

Treatment-resistant schizophrenia (TRS) is a severe form of schizophrenia that has shown poor response to adequate trials of at least two first-line antipsychotics.^1^ TRS places a significant burden on the individuals affected, their caregivers and the healthcare system. In the USA, TRS accounts for 80% of total annual healthcare costs related to schizophrenia.^2^ Approximately one-third of individuals with first-episode schizophrenia already have or go on to develop treatment resistance.^3^ Clozapine is the most effective^4^ and only unanimously guideline-recommended^5^ antipsychotic for TRS. However, its wider use is limited by potentially life-threatening adverse effects, such as ileus,^6^ neutropenia and agranulocytosis,^7^ and myocarditis.^8^ In particular, the risk of clozapine-associated myocarditis (CAM) is underestimated, and this condition may often be overlooked owing to a lack of awareness and knowledge in clinical practice. This is especially problematic given that clozapine has the greatest association with myocarditis among all antipsychotics.^9^ Several mechanisms have been proposed to explain the link between clozapine and myocarditis; these include type-1 hypersensitivity reaction,^10^ but this alone does not fully account for all observed cases. The average onset of CAM is typically around 21 days after initiating treatment,^11^ which suggests that several other mechanisms, such as activation of cardiomyocyte cell death pathways or induction of free radicals and oxidative stress, may also have significant roles.^12^ Emerging evidence supports additional hypotheses including inflammatory or immune-mediated responses involving eosinophilic infiltration, direct myocardial toxicity from clozapine or its metabolites, and cytokine-mediated pathways.^13–16^ To provide a more comprehensive understanding, it is critical to consider these alternative mechanisms while acknowledging the multifactorial nature of CAM. In the context of a potential hypersensitivity reaction, it has been already proposed that CAM might be associated with rapid titration of clozapine.^17^ This is reflected in increasing incidence rates for CAM in countries with guidelines recommending rapid titration, such as Australia,^18^ compared with countries with guidelines proposing slow titration, such as The Netherlands.^17,19^ The target dose after 1 week of clozapine titration varies between 100 mg (Australian national and manufacturing guidelines) and 150 mg (European Summary of Product Characteristics (SmPC) for clozapine), whereas UK community titration schemes suggest a daily dose of 50 mg at day 7^20^ to reduce both side-effect burden and myocarditis risk. International guidance for safer clozapine titration advises a dose between 25 mg and 100 mg at the end of the first week for Asian in-patients with poor clozapine metabolism and European/Western in-patients with average metabolism.^21^ Overall, there is a large body of evidence for complex clozapine-associated inflammatory processes^22^ which might prompt the emergence of CAM.^17^ CAM typically occurs within the first month of antipsychotic treatment.^9^ Important risk factors for the development of CAM include concurrent treatment with sodium valproate^23^; rapid up-titration of clozapine,^24^ potentially inducing inflammation^21^; and altered clozapine metabolism (such as in individuals or Asian or Indigenous American ethnicity or those undergoing concomitant treatment with inhibitors of clozapine metabolism).^24^ Other risk factors include recent infections, history of myocardial infarction, pericarditis, cardiomyopathy and heart failure.^9^ Myocarditis is diagnosed on the basis of clinical presentation, cardiac biomarkers, imaging and histopathology,^25^ with endomyocardial biopsy being the diagnostic gold standard.^26^ Endomyocardial biopsy is generally reserved for cases of suspected myocarditis with ventricular dysfunction and/or heart failure and/or arrhythmia and should not be delayed in hemodynamically unstable presentations.^26^ Patients may present with non-specific symptoms such as flu-like symptoms, fever, fatigue, chest pain or dyspnoea.^27^ When asymptomatic or symptomatic myocarditis occurs in individuals with TRS receiving clozapine, it is crucial to weigh the benefits of continuing clozapine treatment against the risks of discontinuing it, while monitoring for potential signs and complications of myocarditis. High-sensitivity troponin (hs-troponin) testing is generally considered reliable for detection of myocardial injury, as even minimal elevations can indicate cardiac inflammation or damage. However, troponin levels may remain within normal ranges in certain individuals with myocarditis, particularly those with mild cases or those in the early stages.^28^ In such cases, cardiac magnetic resonance imaging (MRI) often plays a critical part in the confirmation of myocarditis, especially when clinical suspicion is high despite normal troponin levels. This is reflected in the modified Lake Louise criteria, which give significant weight to MRI findings.^29^

Once CAM has occurred, clozapine must be discontinued immediately according to the SmPC and should not be offered again as a treatment option. However, re-exposure in cases of CAM can be carried out in selected individuals under close monitoring as part of off-label use.^11,30,31^ Pathophysiological mechanisms for recurrence of CAM during clozapine rechallenge are still not understood, but the risks of recurrence are accepted as being higher during clozapine rechallenge. Currently, there are varying rates of CAM internationally, reflecting differences in both diagnosis and monitoring.^13^ Some countries using active monitoring protocols report higher rates; however, this may also include misdiagnoses and false positives.^32^ Ultimately, overreporting might promote reluctance to prescribe clozapine. Other countries do not monitor at all, leading to underreporting, which is a concern given the high mortality rate of undetected cases.^13,33^

Diagnosing CAM can be challenging owing to its subtle and often non-specific early symptoms, making early detection difficult and sometimes inconsistent across healthcare settings. Current evidence on diagnosis and monitoring of CAM remains limited, and initiation and rechallenge protocols are largely based on case series,^34^ which lack the robustness needed for standardised guidelines. By contrast, cardio-oncology has established clear criteria for diagnosing and managing myocarditis associated with immune checkpoint inhibitors, providing a structured approach grounded in evidence.^35^ Clinical psychiatry would benefit from developing similar criteria for CAM to bring clarity and consistency to clinical decisions where current evidence remains limited. Clear, expert-validated diagnostic criteria are urgently needed to prevent overreporting, ensure accurate diagnosis and ultimately support the broader, safe use of clozapine for individuals with TRS. Furthermore, available CAM protocols have not always included input from cardiology experts, leaving critical gaps in our understanding of the cardiac-specific nuances of this condition and have never yet been comprehensive in terms of covering risk stratification, speed of titration, CAM criteria and monitoring parameters in both clozapine initiation and rechallenge. Thus, we sought to establish a robust multidisciplinary consensus to enhance diagnostic accuracy and refine monitoring recommendations for CAM. This work represents the first Delphi-based international consensus initiative focused on diagnosis, management and rechallenge in cases of CAM.

## Method

### Preparation for the Delphi process

As a first step, we conducted a systematic literature search using the search terms ‘clozapine’ and ‘myocarditis’ on CAM rechallenge cases in PubMed, EMBASE, CINAHL and PsycINFO.^31^ The literature on available CAM protocols was screened and reviewed by the core group (O.D.H., C.U.C., A.H., E.W., D.S., N.K. and M.K.B.).

### Participants and survey structure

We carried out an iterative two-round expert consensus Delphi survey. All members of the Treatment Response and Resistance in Psychosis Working Group (1) were asked via e-mail to contribute as experts and to name a maximum of two cardiologists, two pharmacists and two nurses with clinical or research expertise on clozapine, myocarditis or both. Furthermore, four of the top ten global experts on myocarditis according to Expertscape (https://expertscape.com) search for ‘myocarditis’ on 1 July 2024 were invited to participate in the Delphi; these included authors of international guidelines.^25,26,29^

Then, an online survey was developed and revised by the core group and approved by the local data protection officer and the ethics committee of the medical faculty at LMU Munich, Germany (reference ID: 24-0074KB). The 91 members of the Treatment Response and Resistance in Psychosis Working Group include researchers and clinicians from more than 30 countries across North and South America, Asia, Africa, Oceania and Europe with experience and expertise in the area of schizophrenia.^1^ A Delphi method of consensus development was used, comprising two online survey rounds with licensed software SoSciSurvey (https://www.soscisurvey.de). The first round was conducted on 9 July 2024 and the second round on 2 October 2024. In between, the evidence was synthesised by the core group to develop algorithms. Data on participant professional experience and background were also collected. The complete survey for all Delphi rounds and descriptions of possible ratings appear in the Supplementary Material available at https://doi.org/10.1192/bjp.2025.89. As it is vital that clozapine safety monitoring protocols do not increase burden to patients, we propose various monitoring strategies (minimum versus enhanced) to account for global perspectives and the limited resources in certain healthcare systems, where testing for biomarkers (e.g. NT-proBNP) or imaging facilities (cardiac MRI, echocardiogram) might not always be available.

### Statistical analyses

R 4.4.0 for Windows (R Foundation for Statistical Computing, Vienna, Austria; see https://www.R-project.org/) was used for statistical analyses. Descriptive statistics include frequencies, mean, standard deviation and median. All these parameters were applied to continuous variables, and frequencies were applied to dichotomous variables. In the first round, recommendations with a consensus agreement threshold of ≥75%^36^ were incorporated in the guideline. Recommendations from the first round that reached a subthreshold agreement of >50% were considered again in round 2. Choices from the first round that did not reach consensus were also presented to all experts again. Single statements (e.g. regarding multiple laboratory markers) were synthesised to create the algorithms that were presented in the second round. Here, an agreement threshold of ≥75% was set to define consensus recommendations.

## Results

### Round 1

The results of our systematic search for rechallenge cases and protocols have been published elsewhere^31^ and informed our CAM rechallenge Delphi consensus.

### Characteristics of experts

A total of 93 experts participated in the first round. They had a mean age of 51.7 years (s.d. = 11.6) and mean clinical experience of 23.7 years (s.d. = 11.4); 21.6% of them were female; and they had managed, co-managed or supervised a mean of 472 (s.d. = 672) patients with clozapine since qualification. Fifty-eight (62.4%) were psychiatrists, 14 (15.1%) cardiologists, 12 (14%) pharmacists, one (1%) a psychopharmacologist and one (1%) a nurse, and seven were (7.5%) from research backgrounds; 42.7% of experts were practising in Europe, 22.5% in North America, 15.7% in Asia, 7.9% in Australia/Oceania and in Africa, and 3.4% in South America. Most of the experts reported having witnessed CAM in one in 50 (16.1%), one in 100 (23%) or one in 500 (23%) clozapine-treated cases since qualification (for details for each discipline, see Supplementary Table 1).

### CAM criteria

Most experts (89.6%) judged the modified clinical criteria for immune checkpoint inhibitor-associated myocarditis developed by the European Society of Cardiology (ESC)^35^ to be applicable to CAM (Table 1). The core group agreed on the term CAM instead of clozapine-induced myocarditis, as there is no certainty that all potential cases are clozapine-induced.

**Table 1:** Clinical criteria for clozapine-associated myocarditis (CAM)

CAM: clinical criteria^a^
Clinical diagnosis^b^
Cardiac troponin elevation (new or significant change from baseline)^c^ with one major criterion or two minor criteria, after exclusion of acute coronary syndromes and acute infectious myocarditis based on clinical suspicion
Major criterion
Cardiac magnetic resonance scan that is diagnostic for acute myocarditis (using the modified Lake Louise criteria)^d^
Minor criteria
Clinical syndrome (including any one of the following: fatigue, myalgias, chest pain, diplopia, ptosis, shortness of breath, orthopnoea, lower-extremity oedema, palpitations, light-headedness/dizziness, syncope, muscle weakness, cardiogenic shock)
Ventricular arrhythmia (including cardiac arrest) and/or new conduction system disease
Decline in left ventricular systolic function
Cardiac magnetic resonance imaging scan with features suggestive of myocarditis^e^
Abnormal global longitudinal strain on echocardiography

a Modified based on criteria for immune checkpoint inhibitor-associated myocarditis (ESC 2023 Cardio-Oncology Guidelines)^35^

b Clinical diagnoses should be confirmed with magnetic resonance imaging or pathohistological diagnosis if possible without causing treatment delays.

c Both troponin I and troponin T can be used.

d Lake Louise criteria^29^: T2-based criterion + T1-based criterion + supportive criteria (T2-based criteria: regional or global increase in native T2 or T2 signal intensity; T1-based criteria: regional or global increase in native T1, or regional or global increase in the extracellular volume fraction, or presence of late gadolinium enhancement; supportive criteria: pericarditis and/or regional or global left ventricular systolic dysfunction).

e Suggestive cardiac magnetic resonance scan: meeting some but not all of the modified Lake Louise criteria. The presence of T2- or T1-based criteria may support a diagnosis of acute myocardial inflammation in the appropriate clinical scenario.

### Rechallenge

With respect to rechallenge, 80% (*N* = 70) of the experts believed that rechallenging a patient following an initial episode of CAM could be considered safe, although this perspective does not fully account for complex clinical scenarios, such as cases where patients develop cardiogenic shock or experience ventricular arrhythmias. In such severe presentations, or in situations where cardiac status has not returned to the pre-CAM baseline, rechallenge is not recommended by the core group. Slower up-titration of clozapine in rechallenge, compared with the initiation leading to the index episode of myocarditis, was preferred by 98.6% (*N* = 72) of the group (Supplementary Material).

### Round 2: characteristics of experts

Of the 62 experts who participated in the second round, 42 were psychiatrists, 11 were pharmacists, one was a clinical pharmacologist, five were cardiologists and three were researchers, with a mean age of 52.9 (s.d. = 10) years.

### Algorithm for clozapine initiation (weeks 1–4) with minimum and enhanced monitoring requirements to detect CAM

**Fig. 1:**
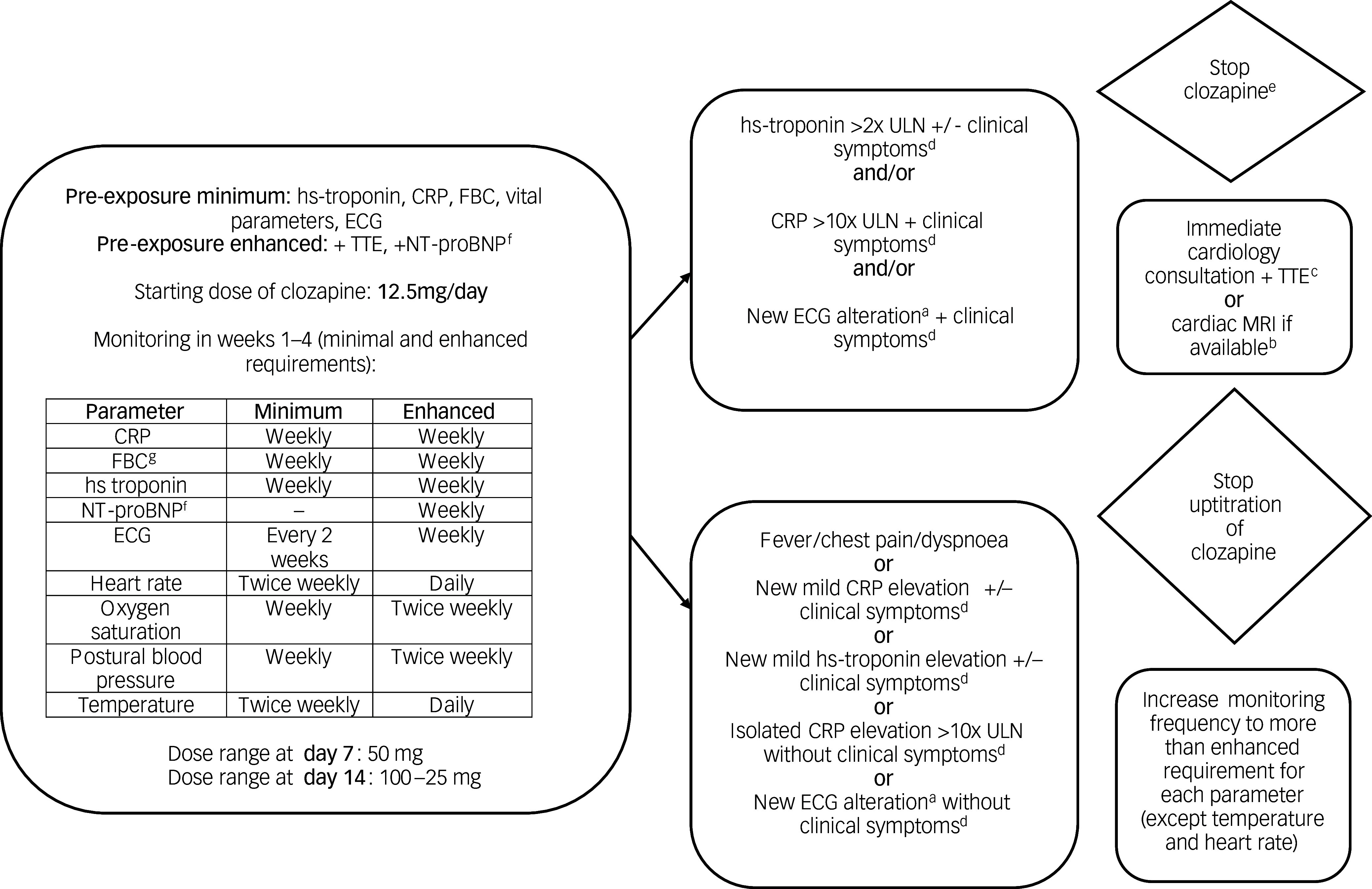
Algorithm for clozapine initiation in weeks 1–4. There was a waiting period of at least 4 weeks after a mRNA vaccination and at least 2–3 weeks (after resolution of the fever related to viral or bacterial infection) to minimise CAM risk. Although the CAM working group has not explicitly endorsed this, the statement implies that mRNA vaccines should not be administered during the 4-week clozapine initiation period. For special requirements, see Table 2. Additional therapeutic drug monitoring including clozapine/norclozapine ratio in week 2 can help to guide understanding regarding potential slow clozapine metabolisers. BP, blood pressure; CRP, c-reactive protein; ECG, electrocardiogram; FBC, full blood count; hs-troponin, high-sensitivity troponin; TTE, transthoracic echocardiography; ULN, upper limit of normal. a. New ST-segment and T-wave deviations. b. Cardiac magnetic resonance imaging (MRI) shall be performed in cases where hs-troponin I/T > 99th percentile, NT-proBNP ≥ 125 pg/mL, or there is a new significant rise from baseline beyond the biological and analytical variation of the assay used. c. Add NT-proBNP if cardiac imaging is not immediately available. d. Clinical symptoms highly suggestive of myocarditis in individuals treated with clozapine are: fever, chest pain or dyspnoea, and flu-like symptoms. e. Potentially life-threatening conditions (such as torsades de points, cardiogenic shock) would trigger clozapine discontinuation before laboratory results are confirmed. f. If available in the respective jurisdiction. g. Only for cardiac monitoring, irrespective of regular absolute neutrophil count (ANC) monitoring which is not covered in this survey.

The algorithm was endorsed by 82.5% (*N* = 57) of experts (Fig. 1). Pre-exposure minimum parameters included hs-troponin, C-reactive protein (CRP), full blood count (FBC), vital parameters and an electrocardiogram (ECG). The starting dose of clozapine was set to 12.5 mg per day. As a minimum requirement, weekly monitoring of hs-troponin, CRP and FBC was recommended in the first 4 weeks. ECG was to be performed every 2 weeks, oxygen saturation (SpO_2_) and postural blood pressure weekly, and heart rate and temperature twice weekly. The majority agreed that additional therapeutic drug monitoring in week 2 (day 14) could help to guide understanding of potential slow clozapine metabolisers (76.9%, *N* = 39).

**Table 2:** Actions and recommendations for specific clinical situations

Drug/condition	Myocarditis risk	Action/recommendation
Modifiable factors		
Valproate co-medication	Increased risk	Do not use if clinically justifiable, or consider slower titration speed and enhanced monitoring
CYP1A2-inhibitors from somatic medicine (e.g. estrogen-containing oral contraceptives)	Faster rise in clozapine levels – possibly increased risk	Consider slower titration speed and enhanced monitoring
Potent CYP1A2-inhibitors from psychiatry (e.g. fluvoxamine)	Faster rise in clozapine levels – possibly increased risk	Do not use if clinically justifiable, or consider slower titration speed and enhanced monitoring
Obesity	Increased risk	Consider slower titration speed and enhanced monitoring
Alcohol misuse	Increased risk	Recommend minimal substance use, or initiate substance use treatment
Intravenous drug use	Increased risk	Recommend minimal substance use, or initiate substance use treatment
Non-modifiable factors		
Asian ancestry or original inhabitants from the Americas	Faster rise in clozapine levels – possibly increased risk	Consider slower titration speed and enhanced monitoring
Chronic infectious diseases (e.g. HIV)	Increased risk	consider slower titration speed and enhanced monitoring
Preceding or concurrent bacterial/viral infection	Increased risk	Consider waiting periods if clinically justifiable and monitor CRP for return to baseline before clozapine initiation if possible, or consider slower titration speed and frequent monitoring
Preceding or concurrent mRNA vaccination	Increased risk	Consider waiting periods if clinically justifiable or consider slower titration speed and frequent monitoring
Pre-existing cardiac condition	Overall increased vulnerability for cardiac adverse drug reactions	Interdisciplinary case discussion, slower titration speed and enhanced monitoring

mRNA, messenger ribonucleic acid; CRP, C-reactive protein; CYP1A2, cytochrome P450 1A2.

To minimise CAM risk, the preferred dose ranges were 50 mg at day 7 (64.5%, *N* = 62), and 100–125 mg at day 14 (67.2%, *N* = 61). Actions and recommendations for specific clinical situations associated with increased myocarditis risk were endorsed by >79.2% of experts (Supplementary Results Q7) and are shown in Table 2. The monitoring algorithm for weeks 1–4 is shown in Fig. 1 and that for weeks 5–8 in Supplementary Table 3 and Supplementary Figs 1 and 2.

### Minimum cardiac monitoring beyond the first 4 weeks

Approximately one-third (30.5%, *N* = 59) suggested extending the monitoring to 8 weeks. If monitoring was continued, vitals (heart rate, oxygen saturation, postural blood pressure and temperature) were to be checked once weekly; and CRP, hs-troponin and FBC (for monitoring of eosinophils) every 2 weeks, with one ECG performed after 4 weeks (agreement of 51.4%, *N* = 35). The monitoring algorithm is shown in Supplementary Table 3.

### Waiting periods after messenger ribonucleic acid (mRNA) vaccination or viral or bacterial infections before clozapine initiation

In the second round, a majority recommended a waiting period of at least 4 weeks after a mRNA vaccination (78%, *N* = 50) and at least 2–3 weeks (89.3%, *N* = 56) after a viral or bacterial infection (after resolution of the fever) to minimise additive CAM risks.

### Algorithm for rechallenge following an episode of CAM with minimum and enhanced requirements

**Fig. 2:**
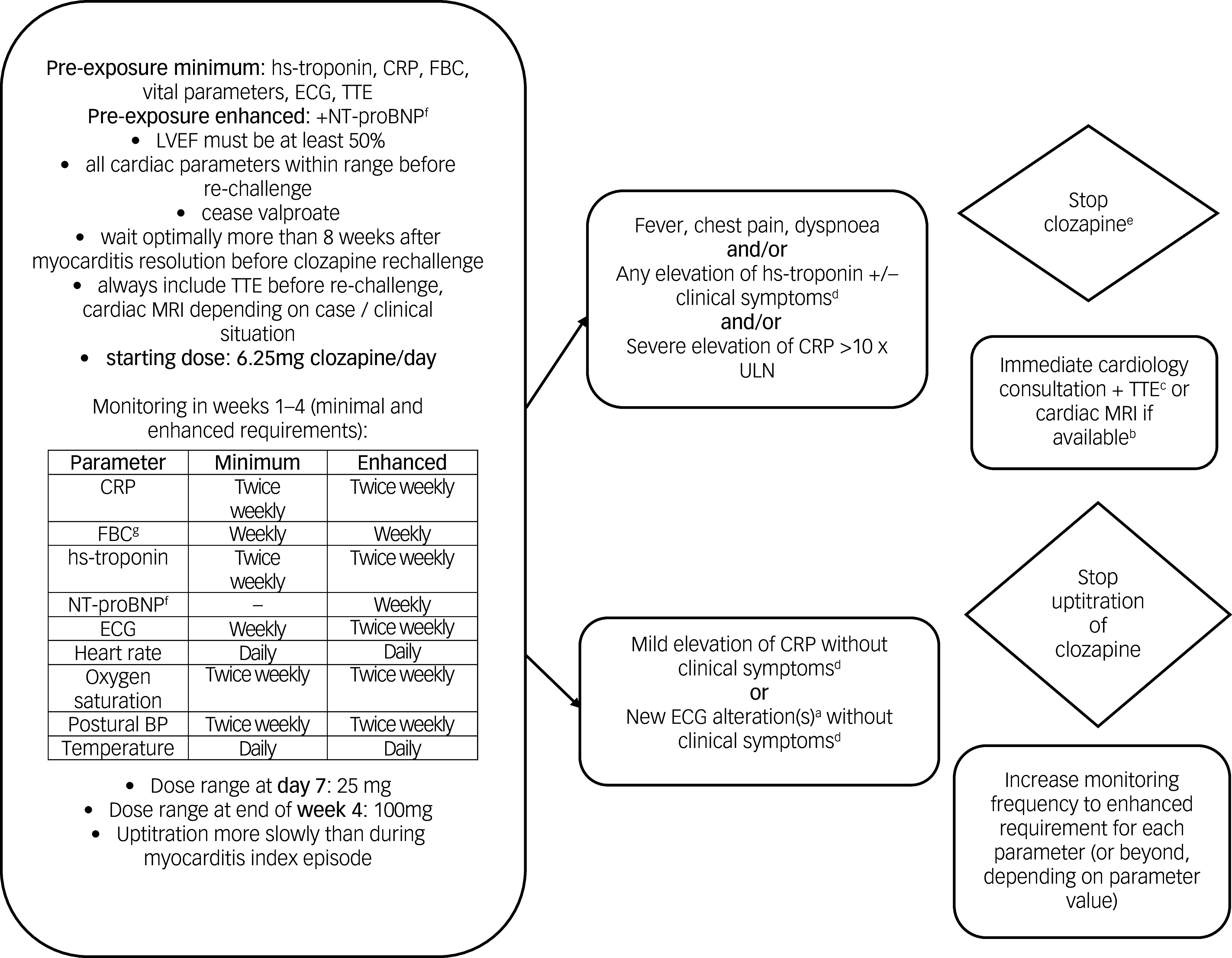
Algorithm for clozapine rechallenge in weeks 1–4. NT-proBNP, N-terminal pro b-type natriuretic peptide; BP, blood pressure; CRP, C-reactive protein; ECG, electrocardiogram; FBC, full blood count; hs-troponin, high-sensitivity troponin; LVEF, left ventricular ejection fraction; TTE, transthoracic echocardiography; ULN, upper limit of normal. a. New ST-segment and T-wave deviations. b. Cardiac magnetic resonance imaging (MRI) shall be performed in cases when hs cardiac troponin I/T >99th percentile, NT-proBN*P* ≥125 pg/mL or new significant rise from baseline beyond the biological and analytical variation of the assay used. c. Add NT-proBNP if cardiac imaging is not immediately available. d. Clinical symptoms highly suggestive of myocarditis in individuals treated with clozapine are: fever, chest pain or dyspnoea, and flu-like symptoms. e. Potentially life-threatening conditions (such as torsades de points, cardiogenic shock etc.) would trigger discontinuation before laboratory results are confirmed. f. If available in the respective jurisdiction. g. Only for cardiac monitoring, irrespective of regular absolute neutrophil count (ANC) monitoring which is not covered in this survey.

The core group agreed that for rechallenge, an informed consent process for off-label use of clozapine should be recommended. The developed algorithm was endorsed by 87.3% (*N* = 55) of experts (Fig. 2).

Before clozapine rechallenge, recommended minimum investigations included hs-troponin, CRP, FBC, vital parameters, ECG and transthoracic echocardiography with a left ventricular ejection fraction of at least 50%. Enhanced investigations included additional testing for NT-proBNP. All minimum requirement parameters were to be within range before rechallenge, and sodium valproate was to be ceased if clinically justifiable before the rechallenge trial. A minimum waiting period of 8 weeks after resolution of myocarditis, where possible, was recommended. As a minimum requirement, twice-weekly monitoring of hs-troponin and CRP and weekly monitoring of FBC was recommended. ECG was to be performed weekly, oxygen saturation and postural BP measured twice weekly, and heart rate and temperature measured daily.

To minimise recurrence of myocarditis, the preferred doses were a 6.25 mg starting dose at day 1 (94.7%, *N* = 57), 25 mg at day 7 (91.1%, *N* = 56), 50 mg at day 14 (90.7%, *N* = 54) and 100 mg at the end of week 4 (88.7%, *N* = 53). In the absence of complications, the protocol could be stepped down to weekly biomarker and twice weekly vital monitoring in weeks 5–8 (91.4%, *N* = 58). The algorithm for weeks 1–4 is shown in Fig. 2 and that for weeks 5–8 in Supplementary Table 5 and Supplementary Figs 3 and 4.

## Discussion

This is the first international and multidisciplinary Delphi consensus project among experts on clozapine or myocarditis to synthesise evidence generated from a systematic literature search and evaluation and real-world-experience of clozapine initiation and rechallenge. Previous monitoring or rechallenge protocols for CAM have mostly been derived from case reports, case series and expert opinions,^28,34,37^ rather than robust, consensus-based frameworks, and only three CAM protocols have been developed involving both psychiatrists and cardiologists.^13,34,37^ Furthermore, the variability in recommendations (e.g. with respect to timing and duration of monitoring) derived from current CAM protocols can lead to inconsistency in clinical practice and complicate efforts to identify myocarditis early, potentially leading to either under- or overreporting of CAM.

In a meta-analysis by Siskind et al, an event rate of 0.7% was reported for CAM.^8^ Recently an incidence of 8.5% CAM risk was reported in a part of Australia employing a mandatory screening programme for CAM.^32^ Reported mortality rates after CAM vary between 21 and 50%.^11,38^ There may be significant underreporting of cases in countries where CAM is not actively monitored, but overreporting is also a consideration, as an Australian chart review found that 13 of 20 (65%) cases did not meet the ‘Ronaldson criteria’ for a clinical diagnosis of CAM.^39^ Here, although elevated troponin was present in 17 of 20 (85%) cases, other parameters were either not assessed or not indicative of myocarditis.^39^ In a retrospective record database analysis from the UK, among 228 patients with suspected CAM, only 11.4% were confirmed as having probable CAM; this highlights the need for clear CAM criteria.^40^ As a central outcome of our project, we established clear criteria for CAM based on guidelines for myocarditis related to immune checkpoint inhibitor treatment. This could help to reduce overreporting, as, according to expert consensus, confirmation of CAM now requires mandatory use of at least one cardiac imaging technique, such as echocardiography or cardiac MRI. Transthoracic echocardiography is far more available globally, making it a more pragmatic choice in many settings, even though it may lack the detailed diagnostic precision of cardiac MRI. Another significant finding of our consensus Delphi was the critical importance of slow clozapine titration; this is supported by the fact that countries where slower titration in out-patient settings is widely practised, including Denmark, The Netherlands and the UK,have reported low rates of CAM.^41–44^ This suggests that the titration scheme proposed by the clozapine manufacturer may be too rapid. Our consensus also confirmed the diagnostic value of hs-troponin and CRP testing in screening for CAM, whereas certain clinical symptoms including sinus tachycardia were considered to be of minor diagnostic value.^40^ Nevertheless, sinus tachycardia may be both a transient phenomenon during clozapine up-titration and a non-specific marker of potential CAM.^28^ Guidelines from Denmark and The Netherlands do not consider CRP or hs-troponin screening to be mandatory^45^ and only recommend testing these parameters as part of a diagnostic evaluation when CAM is suspected.^45^ There is no mandatory screening or monitoring guideline for CAM in the SmPC; this means that the protocol of Ronaldson et al, which does specify monitoring parameters but not clozapine titration speed, is adopted in most cases.^28^

Guidelines in Australia, New Zealand and Germany require mandatory weekly CRP and troponin assessments during the first 4–6 weeks.^46^ One previously published protocol suggested weekly monitoring of cardiac troponin, NT-proBNP and CRP for a total duration of 8 weeks,^34^ whereas our core recommendation is for 4–8 weeks with reduced monitoring frequency in the last 4 weeks; this underscores the necessity for close monitoring in the first month, in which most CAM cases occur according to the literature.^9^ It is also important to consider potential false-positive elevations in troponin levels, such as those caused by heterophile antibodies in infectious mononucleosis, fibrin clots, renal dysfunction, pulmonary embolism or rheumatoid factor.^47,48^ Other causes of elevated troponin, including sepsis and renal impairment, should also be thoroughly evaluated to avoid misdiagnosis. Moreover, there is considerable variability in the sensitivity of troponin platforms, with variations possible even within the same pathology laboratory; thus, using a threshold of twice the upper limit of normal could lead to false-positive troponin results. To reduce false positives, it may be beneficial to implement local cut-offs for (a) acceptable minor elevation, (b) elevation that warrants repeat testing and (c) clear elevation^49^ in the context of concurrent causes of elevated troponin. CRP alone has been suggested as an effective screening marker for inflammation in the absence of genetic testing for clozapine poor-metaboliser status,^21^ as its levels often rise several days before troponin,^32,50^ potentially reducing the need for routine troponin assessments.^21,51^ Moreover, the literature describes cases of CAM in which CRP elevations occur without concurrent troponin elevation,^28^ a notably rare scenario when using hs-troponin. Nevertheless, although CRP elevation indicates inflammation, it does not reliably demonstrate cardiac involvement, making hs-troponin essential for accurate identification of early myocardial changes associated with clozapine use. This can be juxtaposed with a strategy in which troponin testing is only necessary as confirmation of emerging cardiac issues in suspected CAM cases.^32^ Hs-troponin should be incorporated into weekly screening protocols during the first 4 weeks of clozapine treatment, a period of heightened myocarditis risk, owing to its specificity in detecting myocardial injury. Unlike CRP, which is susceptible to fluctuations from various non-cardiac factors, such as infections, smoking and systemic inflammatory conditions, hs-troponin specifically indicates cardiac injury, making it a more reliable marker in this context. CRP elevations can be misleading, reflecting general inflammatory responses rather than myocarditis, and thus may obscure early detection of myocardial involvement. Routine weekly hs-troponin assessments in the initial treatment phase provide a targeted and consistent approach to monitoring, enabling prompt intervention if myocarditis develops.

According to our expert panel, hs-troponin as a singular cardiac laboratory marker is sufficient for effective CAM screening, with NT-proBNP testing reserved for an enhanced monitoring scheme. This approach reduces unnecessary barriers to clozapine use by simplifying screening requirements without compromising patient safety. As genetic testing is not anchored in clinical practice, the issue of incorrect self-identification and mixed ancestries must be mentioned when suggesting slower titration schemes or lower target clozapine doses for individuals of a specific ancestry. Nevertheless, on the basis of slow clozapine metabolism in people with Asian ancestry,^52,53^ a maximum titration rate of 25 mg per week for individuals of Japanese ancestry has been proposed,^52,54^ and the available evidence regarding slower metabolisers should be taken into account to reduce CAM risk.

Although the role of eosinophils in CAM diagnosis has been debated over time,^14,28^ we also included FBC, as it has previously been suggested that monocytosis may be an early marker of CAM, whereas eosinophilia may be a late marker of a hypersensitivity reaction.^37^ Ultimately, both of these alterations should prompt enhanced CAM monitoring. Eosinophilia typically occurs after troponin elevation,^55^ which has led to the exclusion of eosinophils in earlier monitoring protocols^28^; however, the assessment does not impose an additional burden in the context of mandatory weekly absolute neutrophil count monitoring during the period of CAM monitoring, and the proportion of CAM survivors with eosinophilia has been reported to be as high as 66%.^55^ Repeated ECG monitoring was endorsed, similar to previous CAM protocols developed by multidisciplinary working groups,^34^ as it is considered to be usually abnormal in myocarditis, although ECG signs are neither specific nor sensitive,^25^ and it is recommended in all individuals with clinically suspected myocarditis.^25^

In the previous Australian CAM monitoring protocol from Ronaldson and colleagues, baseline transthoracic echocardiography before commencing clozapine was presumed to be clinically warranted. This would mean that myocarditis, when suspected, could be diagnosed with a high degree of specificity, and appropriate treatment could be established for any cardiac disease detected.^28,56^ Nevertheless, this was not recommended in later CAM protocols,^34^ as from a global and more pragmatic perspective, implementing mandatory baseline echocardiography (defined as a minimal requirement in our consensus) would probably result in fewer individuals with TRS receiving clozapine in healthcare systems where echocardiography is unavailable. In such contexts, in the trade-off between benefits and risks, our experts prioritised close cardiac monitoring during treatment over achieving highly refined risk stratification at baseline, and so baseline echocardiography was only recommended in the enhanced monitoring investigations. By contrast, baseline echocardiography was considered to be mandatory before potential clozapine rechallenge; this is in line with previous rechallenge protocols^13,34,37^ and acknowledges that rechallenge should not be recommended in cases of cardiomyopathy with significantly reduced left ventricular ejection fraction. Whereas previous guidelines recommended against rechallenge,^57^ this study found that the majority of experts approved rechallenge in selected cases after CAM. It is important to caution that clozapine rechallenge after CAM is a multiprofessional clinical decision involving psychiatry, cardiology and pharmacy that has to be carefully considered in terms of risks versus benefits together with the patient and their family.

A previous CAM protocol recommended a waiting period of at least 6 months after an episode of CAM to allow the inflammation to resolve and the myocardium to recover before rechallenge,^34^ whereas in our Delphi survey, a minimum waiting period of 8 weeks was considered adequate. The limited evidence regarding rechallenge after myocarditis related to immune checkpoint inhibitor therapy indicates that clozapine rechallenge should be a multidisciplinary decision and considered on an individual basis. For example, patients with a mild CAM index episode might be considered for earlier rechallenge (minimum waiting period of 8 weeks), whereas those with more severe CAM may require a longer waiting period, often at least 6 months, as proposed by Griffin et al.^34^ According to the expert panel, the shorter timeframe for individuals with a mild index episode allows clinicians to safely monitor resolution of inflammation while minimising the risk of inadequately treated psychosis.

Our algorithms will provide guidance for management of selected cases in which rechallenge is considered to be a viable option. The recommendations in this consensus statement should be validated using prospective CAM registry-based studies^31^ with standardised interventions and outcome measurements. The results of our Delphi process can be regarded to represent a consensus guideline, as we used a structured methodology to gather expert opinions and progressively refine them towards a common agreement. This process is particularly valuable in fields such as CAM, in which empirical evidence may be limited, but expert clinical experience and judgement are critical. By incorporating a diverse range of expert insights and iterating through multiple rounds, the Delphi method ensures that the final guidelines represent a broad-based consensus that is both robust and well informed, resulting in a reliable framework for clinical decision-making. In summary, our multidisciplinary Delphi process represents the most representative guideline-related endeavour regarding prevention, screening and monitoring of CAM to date, and its results may inform clinicians and future guidelines.

## Supporting information

Wagner et al. supplementary material 1 Wagner et al. supplementary material

Wagner et al. supplementary material 2 Wagner et al. supplementary material

Wagner et al. supplementary material 3 Wagner et al. supplementary material

Wagner et al. supplementary material 4 Wagner et al. supplementary material

## Data Availability

Data are available upon reasonable request.

## Supplementary material

The supplementary material is available online at https://doi.org/10.1192/bjp.2025.89
