# Multidisciplinary consensus on prevention, screening and monitoring of clozapine-associated myocarditis and clozapine rechallenge after myocarditis – ADDENDUM

**DOI:** 10.1192/bjp.2025.10372

**Published:** 2026-04

**Authors:** Elias Wagner, Nicole Korman, Marco Solmi, Matin Mortazavi, Zahra Aminifarsani, Douglas Dubrovin Leão, Matthew K. Burrage, Dan Siskind, Laura McMahon, Oliver D. Howes, Christoph U. Correll, CAM Expert Group, Alkomiet Hasan

Supplementary material was omitted from the original publication. The file ‘CAM Expert Group’ has been added as supplementary material.
