# Supplementary material for: Multidisciplinary consensus on prevention, screening and monitoring of clozapine-associated myocarditis and clozapine rechallenge after myocarditis
Source: Br J Psychiatry. 2025 Jun 2;228(4):348–56. doi: 10.1192/bjp.2025.89 (PMC13051212; doi:10.1192/bjp.2025.89)
Supplement: Wagner et al. supplementary material 2 — Wagner et al. supplementary material [file S0007125025000893sup002.pdf]

Clozapine rechallenge (week 1-4)

- **Pre-exposure minimum:** high-sensitivity troponin, CRP, FBC, vital parameters, ECG, TTE (LVEF must be ≥50%), cardiac MRI depending on case/clinical situation
- Pre-exposure mimimum parameters should be within range before re-challenging and Sodium Valproate should be ceased if clinically justifiable
- Wait optimally more than 8 weeks after myocarditis resolution before rechallenge, all cardiac lab results must be within range before rechallenge, uptitration more slowly than during myocarditis index episode
- Caveat: Shared-decision making process weighing benefits vs. risks (off-label use)
- **Pre-exposure enhanced:** + NT-proBNP[1]
- **Starting dose of clozapine:** 6.25 mg

Minimum and enhanced monitoring requirements in weeks 1-4 (clozapine rechallenge)

	Heart rate	Temperature	SpO_2	ECG	postural BP	hs troponin	CRP	NT-proBNP[1]	FBC[2]
	Clinical			Instrumental		Laboratory parameters			
Parameter	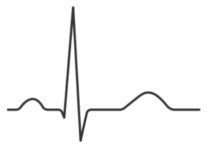	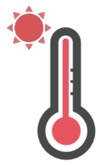	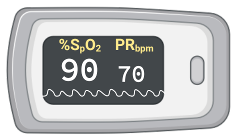	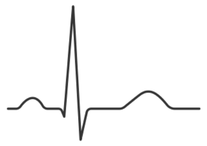	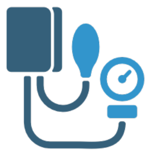	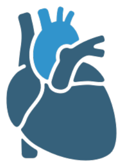	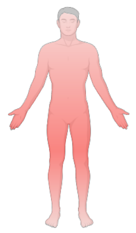	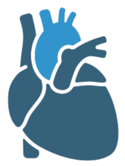	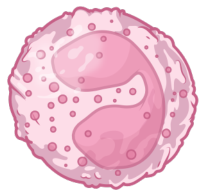
Minimum	daily	daily	twice weekly	weekly	twice weekly	twice weekly	twice weekly	-	weekly
Enhanced	daily	daily	twice weekly	twice weekly	twice weekly	twice weekly	twice weekly	weekly	weekly

Dose range for clozapine at **day 7**: 25mg

Dose range for clozapine at **end of week 4**: 100mg

Supplementary Table 4: Minimum and enhanced monitoring requirements in weeks 1-4 (clozapine rechallenge) Created with biorender

Minimum and enhanced monitoring requirements in weeks 5-8 (clozapine rechallenge)

	Heart rate	Temperature	SpO_2	ECG	postural BP	hs troponin	CRP	NT-proBNP[1]	FBC[2]
	Clinical			Instrumental		Laboratory parameters			
**Parameter**	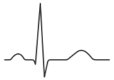	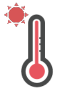	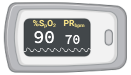	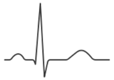	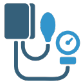	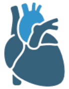	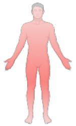	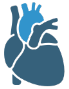	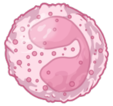
**Minimum**	twice weekly	twice weekly	weekly	every 2 weeks	twice weekly	weekly	weekly	-	weekly
**Enhanced**	daily	daily	twice weekly	twice weekly	twice weekly	twice weekly	twice weekly	weekly	weekly

Legend

[1] if available in the respective jurisdiction

[2] only for cardiac monitoring irrespective of regular ANC monitoring

[3] clinical symptoms highly suggestive of myocarditis in individuals treated with clozapine are: fever, chest pain or dyspnoea and flu-like symptoms

[4] i.e. new ST-segment and T-wave deviations

[5] potentially life-threatening conditions (such as torsades de points, cardiogenic shock, etc) would trigger clozapine discontinuation before laboratory results are confirmed

[6] add NT-proBNP if cardiac imaging is not immediately available

[7] cardiac MRI shall be performed in cases when hs troponin I/T > 99th percentile, NT-proBNP ≥ 125 pg/ml or new significant rise from baseline beyond the biological and analytical variation of the assay used.

Supplementary Table 5: Minimum and enhanced monitoring requirements in weeks 5-8 (clozapine rechallenge) Created with biorender

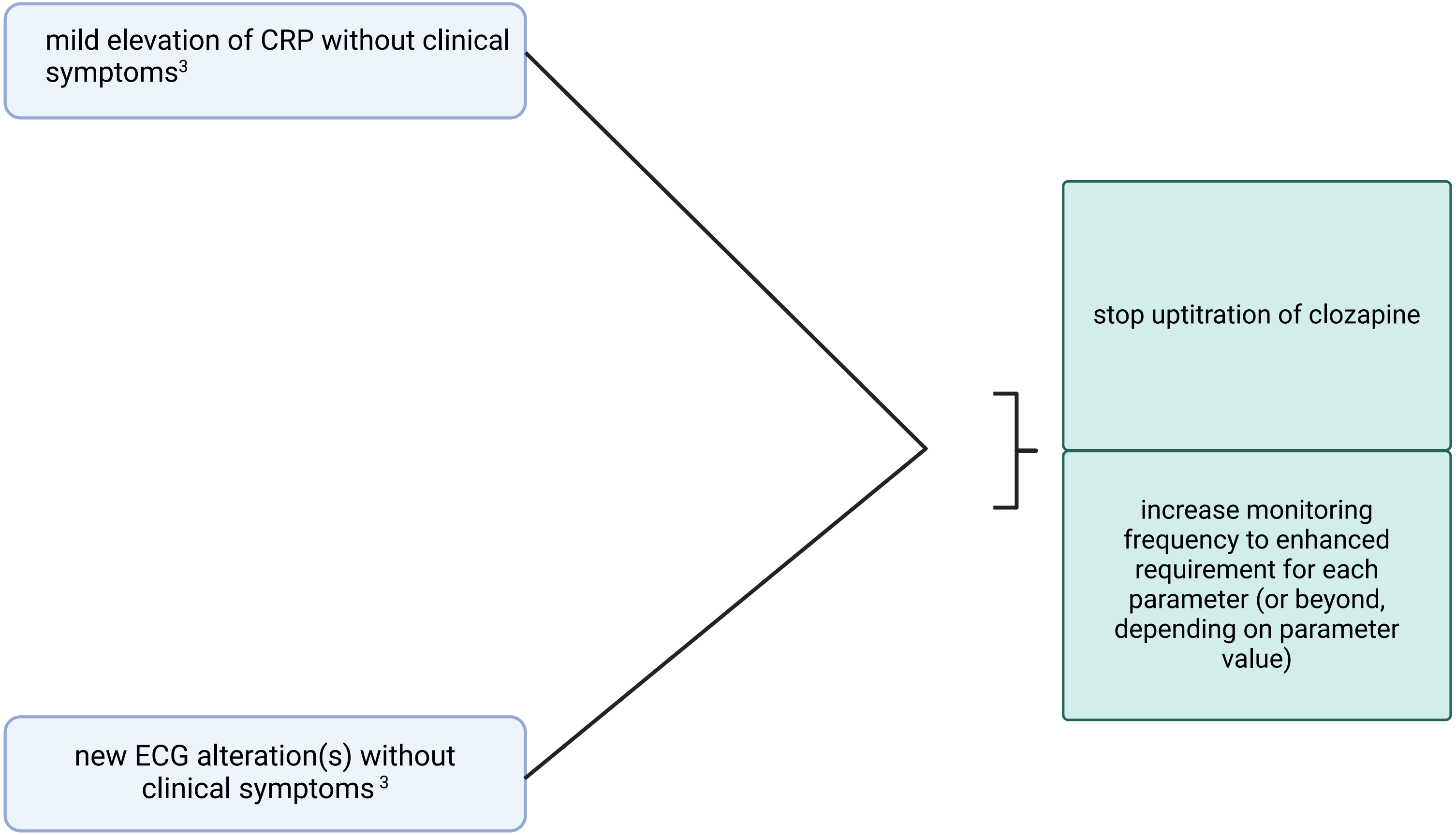

General rules:

- Caveat: special requirements (see Table 2)

Supplementary Figure 3: Algorithm for monitoring during clozapine rechallenge. Created with biorender

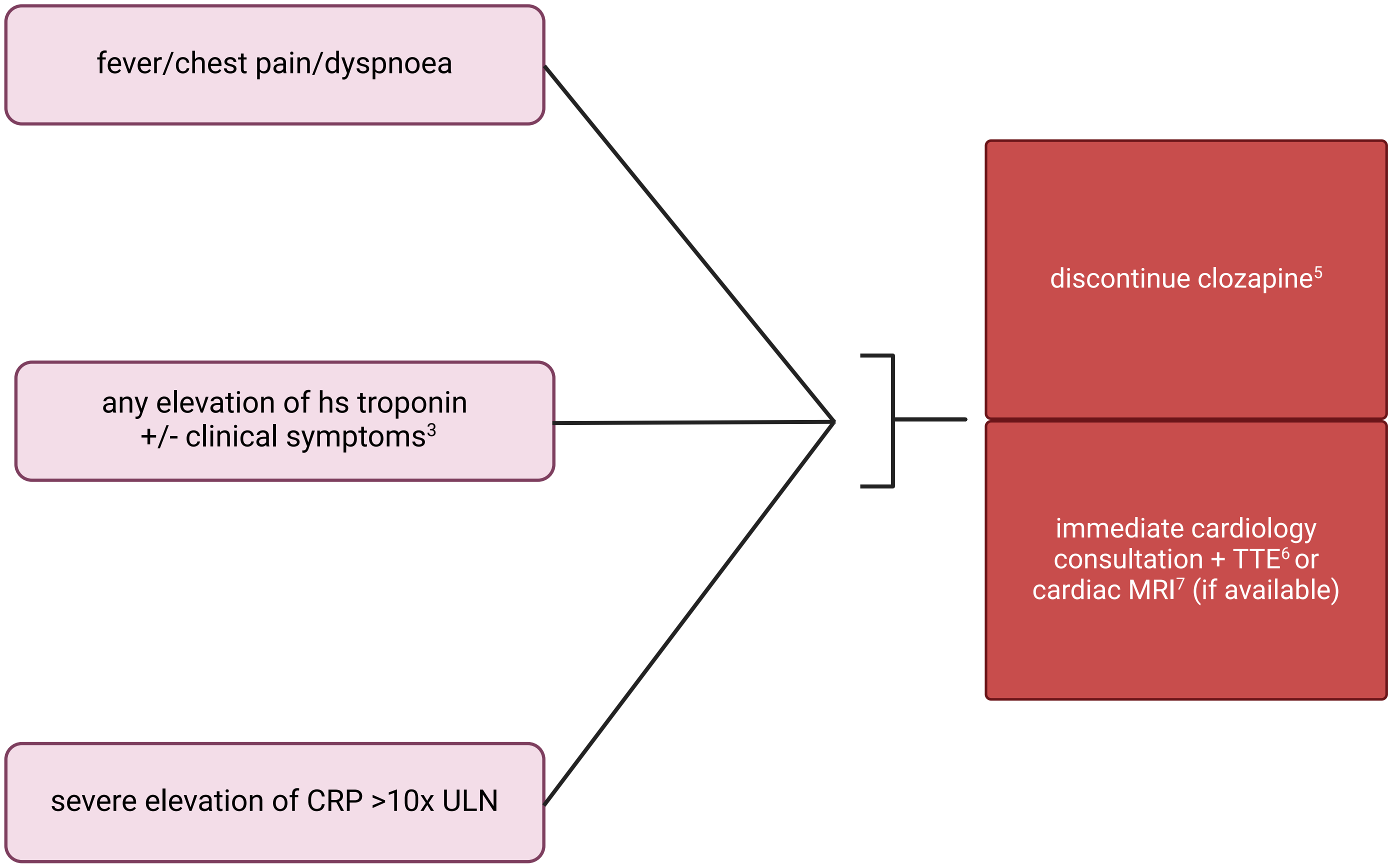

Supplementary Figure 4: Algorithm for discontinuation during clozapine rechallenge. Created with biorender
