# Supplementary material for: Multidisciplinary consensus on prevention, screening and monitoring of clozapine-associated myocarditis and clozapine rechallenge after myocarditis
Source: Br J Psychiatry. 2025 Jun 2;228(4):348–56. doi: 10.1192/bjp.2025.89 (PMC13051212; doi:10.1192/bjp.2025.89)
Supplement: Wagner et al. supplementary material 3 — Wagner et al. supplementary material [file S0007125025000893sup003.pdf]

# *Supplementary Material*

## *Multidisciplinary consensus on prevention, screening and monitoring of clozapine-associated myocarditis and clozapine rechallenge after myocarditis*

*Elias Wagner[1,2], Nicole Korman[3,4,5], Marco Solmi[6], Matin Mortazavi[1,2], Zahra Aminifarsani[1,2], Douglas Silva Leao[2], Matthew K Burrage[7,8], Dan Siskind[3,4,5,9], Laura McMahon[6,10], Oliver Howes[11], Christoph U Correll[12,13,14], CAM expert group*, Alkomiet Hasan[2,15]*

[1]Evidence-based Psychiatry and Psychotherapy, Faculty of Medicine, University of Augsburg, Augsburg, Germany.

[2]Department of Psychiatry, Psychotherapy and Psychosomatics, Medical Faculty, University of Augsburg, BKH Augsburg, Augsburg, Germany.

[3]Queensland Centre for Mental Health Research, Wacol, Queensland, Australia.

[4]Metro South Addiction and Mental Health Service, Metro South Health, Brisbane, Brisbane South, Queensland, Australia.

[5]University of Queensland School of Clinical Medicine, Brisbane, Queensland, Australia.

[6]Department of Psychiatry and Ottawa Hospital Research Institute Clinical Epidemiology Program, University of Ottawa, Ottawa, ON, Canada; Regional Centre for the Treatment of Eating Disorders and On Track: The Champlain First Episode Psychosis Program, Department of Mental Health, The Ottawa Hospital, Ottawa, ON, Canada; Department of Child and Adolescent Psychiatry, Charité Universitätsmedizin, Berlin, Germany.

[7]Faculty of Medicine, University of Queensland, Brisbane, Australia.

[8]Department of Cardiology, Ipswich Hospital, Queensland, Australia.

[9]Queensland Brain Institute, University of Queensland, Brisbane, Queensland, Australia.

[10] Mental Health Service, Darling Downs Health District, Toowoomba, Australia

[11]Institute of Psychiatry, Psychology and Neuroscience, King's College London, London, UK.

[12]Department of Psychiatry, Zucker Hillside Hospital, Glen Oaks, NY, USA

[13]Departments of Psychiatry and Molecular Medicine, Donald and Barbara Zucker School of Medicine at Hofstra/Northwell, Hempstead, NY, USA

[14]Department of Child and Adolescent Psychiatry, Charité Universitätsmedizin Berlin, Berlin, Germany

[15]DZPG (German Center for Mental Health), partner site München/Augsburg, Augsburg, Germany

**#Corresponding author**:

Prof. Dr. med. Elias Wagner

Address: Department of Psychiatry, Psychotherapy and Psychosomatics, Medical Faculty, University of Augsburg, BKH Augsburg, Augsburg, Germany

e-mail: elias.wagner@med.uni-augsburg.de

Running title:

Keywords (max. 6): clozapine, myocarditis, cardio-toxicity, schizophrenia, treatment-resistance

## Supplementary Results

| Profession | N total | Sex (Female/Male) | Age (Age range, Mean ± SD, N) | Continent of practice (ratio, N) | Clinical experience in years (Mean ± SD, N) | Number of clozapine patients (co-) managed or supervised (Mean ± SD, N) | Patients with CAM witnessed (ratio, N) |
|---|---|---|---|---|---|---|---|
| *All* | 93 | Female: 20 (21.6%)<br>Male: 73 (78.4%) | 46, 51.71 ± 11.62, 93 | Europe: 42.7%, 38<br>North America: 22.5%, 20<br>South America: 3.4%, 3<br>Africa: 7.9%, 7<br>Asia: 15.7%, 14<br>Australia/Oceania: 7.9%, 7 | 23.68 ± 11.37, 81 | 472.22 ± 671.68, 89 | 1 in 10: 4.6%, 4<br>1 in 25: 6.9%, 6<br>1 in 50: 16.1%, 14<br>1 in 100: 23.0%, 20<br>1 in 500: 23.0%, 20<br>Less than 1 in 500: 26.4%, 23 |
| *Psychiatrists* | 58 | Female: 6 (10.3%)<br>Male: 52 (89.7%) | 44, 52.86 ± 10.82, 58 | Europe: 34.5%, 20<br>North America: 20.7%, 12<br>South America: 5.2%, 3<br>Africa: 10.3%, 6<br>Asia: 24.1%, 14<br>Australia/Oceania: 5.2%, 2 | 24.09 ± 10.52, 53 | 557.83 ± 735.32, 58 | 1 in 10: 6.9%, 4<br>1 in 25: 6.9%, 4<br>1 in 50: 15.5%, 9<br>1 in 100: 27.6%, 16<br>1 in 500: 15.5%, 9<br>Less than 1 in 500: 27.6%, 16 |
| *Cardiologists* | 14 | Female: 4 (28.6%)<br>Male: 10 (71.4%) | 34, 52.36 ± 12.09, 14 | Europe: 53.8%, 7<br>North America: 30.8%, 4<br>South America: -<br>Africa: 7.7%, 1<br>Asia: -<br>Australia/Oceania: 7.7%, 1 | 26.42 ± 12.43, 12 | 76.08 ± 134.58, 13 | 1 in 10: -<br>1 in 25: 8.3%, 1<br>1 in 50: 16.7%, 2<br>1 in 100: 16.7%, 2<br>1 in 500: 25.0%, 4<br>Less than 1 in 500: 33.3%, 4 |
| *Pharmacists* | 13 | Female: 6 (46.2%)<br>Male: 7 (53.8%) | 40, 51.15 ± 12.90, 13 | Europe: 50.0%, 6<br>North America: 25.0%, 3<br>South America: -<br>Africa: -<br>Asia: -<br>Australia/Oceania: 25.0%, 3 | 23.36 ± 11.25, 11 | 420.83 ± 426.65, 12 | 1 in 10: -<br>1 in 25: -<br>1 in 50: 25.0%, 3<br>1 in 100: 8.3%, 1<br>1 in 500: 41.7%, 5<br>Less than 1 in 500: 25.0%, 3 |
| *Nurses* | 1 | Female: 1 (100%) | 54, 1 | North America:100.0%, 1 | 33, 1 | 1500, 1 | Missing-No answer |

| | | | | | | | |
|---|---|---|---|---|---|---|---|
| *Others* | 7 | Female: 3 (42.9%)<br>Male: 4 (57.1%) | 35, 41.57 ± 13.24, 7 | Europe: 100.0%, 5<br>North America: -<br>South America: -<br>Africa: -<br>Asia: -<br>Australia/Oceania: - | 8.50 ± 13.08, 4 | 427.00 ± 880.17, 5 | 1 in 10: -<br>1 in 25: 20.0%, 1<br>1 in 50: -<br>1 in 100: 20.0%, 1<br>1 in 500: 60.0%, 3<br>Less than 1 in 500: - |

**Supplementary Table 1**. Demographic information of experts based on profession.

## Results from Round 2:

### Q1

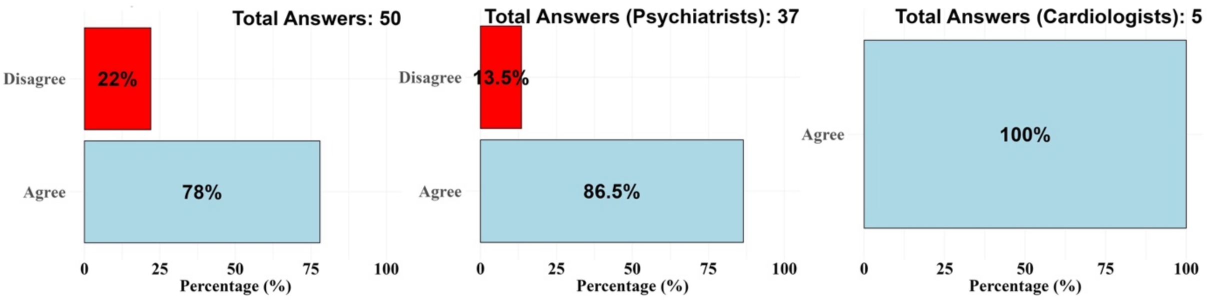


A majority (55.6%) recommended a waiting period after an mRNA vaccination before clozapine initiation. The average waiting period was <u>at least 4 weeks</u>. Would you agree? Please rate with agree or not agree or no answer.

### Q2

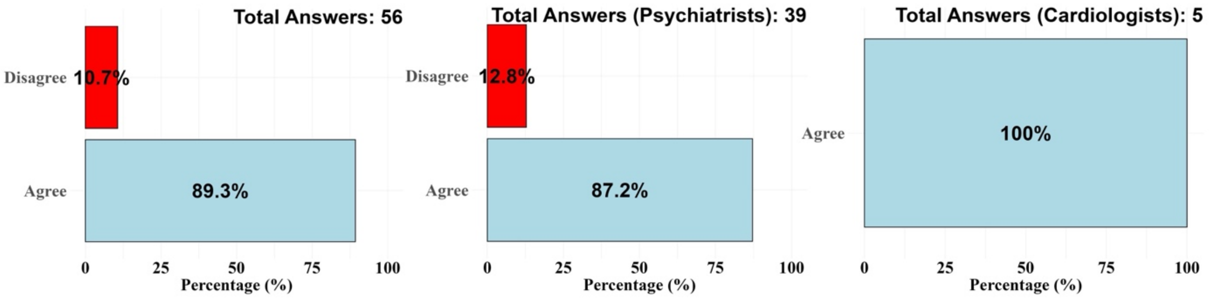


A majority (70.5%) recommended a waiting period after a viral or bacterial infection (after resolution of the fever) before clozapine initiation. The average waiting period was <u>at least 2-3 weeks (2.5 weeks).</u> Would you agree? Please rate with agree or not agree or no answer.

### Q3

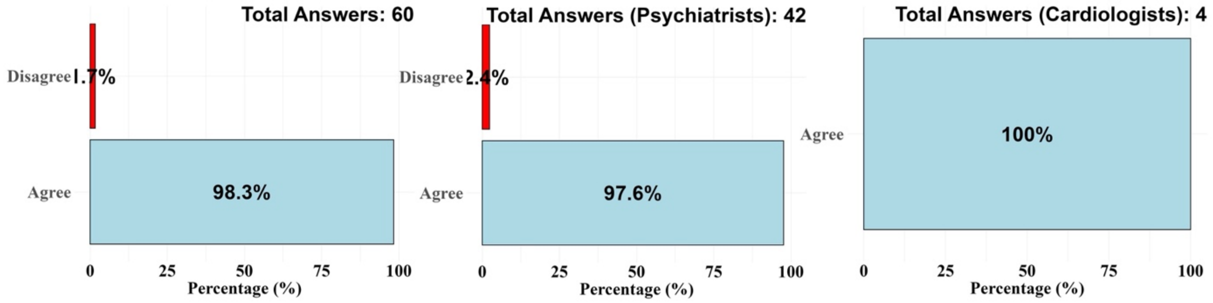


Since some populations will need lower doses to reach concentrations of 350 ng/ml (i.e. female, non-smoker, East Asian / Native American, higher BMI, concurrent inflammation) compared to others, identifying these poor metabolizers to guide titration is crucial. For these populations, slower titration schemes (as compared to general) are recommended in the literature (De Leon et al., 2021). Please rate with agree / not agree / no answer.

**Q4**

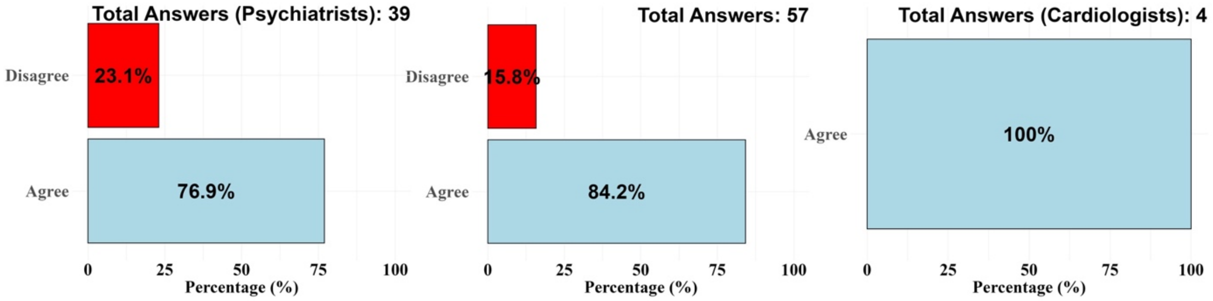


Do you think drawing clozapine levels in week 2 (~day 14) can help to guide understanding around potential slow metabolisers? Please rate with agree / not agree / no answer.

**Q5**

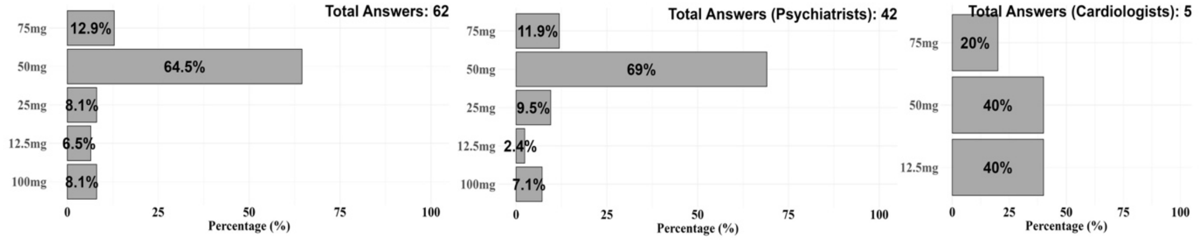


There appears to be a link between faster speed of clozapine titration and higher rates of myocarditis (Carswell et al., A 12-month audit of clozapine associated myocarditis in a South Australian Local Health Network: The importance of screening and personalised titration. *Schizophrenia research*, 2023). Some guidelines suggest that for people WITHOUT special conditions mentioned above (eg slow metabolisers) titration should reach 100mg (Australian national and manufacturing guideline) or 150mg at day 7 (i.e. European SmPC of clozapine). However, this speed of titration appears to increase the risk of myocarditis (Carswell et al., Schizophrenia research 2023). By contrast, for community clozapine titration in the UK, for example, a daily dose of 50mg at day 7 was recommended (Beck et al., Acta Psychiatrica Scandinavica 2014) to reduce side-effect burden, including myocarditis risk.
If we were to recommend a titration schedule that potentially reduces the risk of myocarditis, what would be the recommended daily dose of clozapine at day 7?

**Q6**

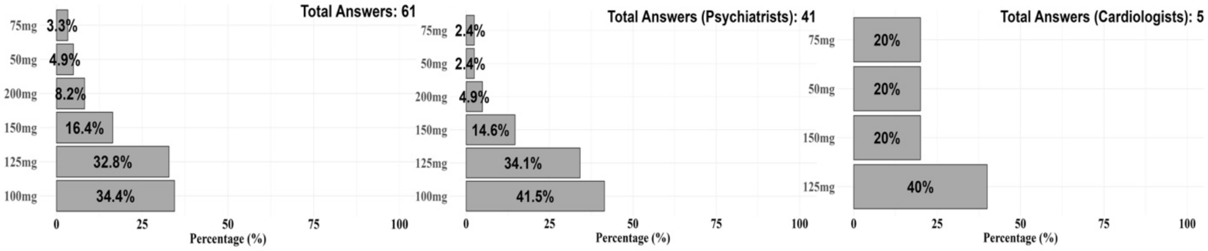


In the same context as in the previous question, some guidelines suggest that for people WITHOUT special conditions mentioned above (eg slow metabolisers) titration should reach 200mg (Australian national and manufacturing guideline) or 300 mg by day 14 (i.e. European SmPC of clozapine). However, this speed of titration appears to increase the risk of myocarditis (Carswell et al., Schizophrenia research 2023). By contrast, for community clozapine titration in the UK, for example, a daily dose of 125 mg at day 14 was recommended (Beck et al., Acta Psychiatrica Scandinavica 2014) to reduce side-effect burden, including myocarditis risk. If we were to recommend a titration schedule that potentially reduces the risk of myocarditis, what would be the recommended daily dose of clozapine at day 14?

**Q7a**

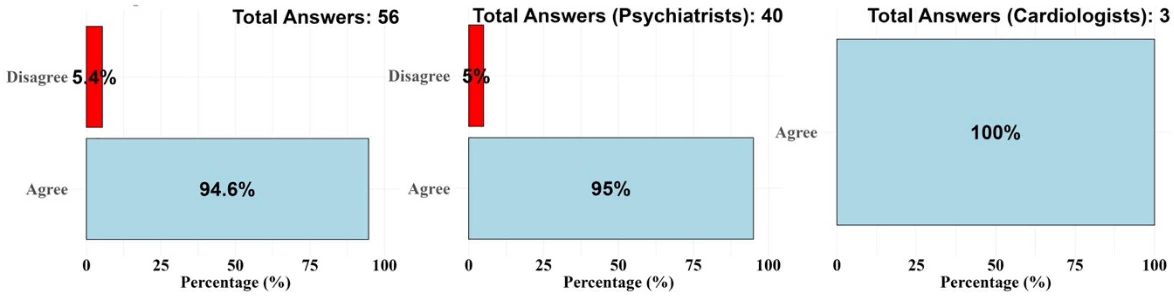


Not only a more rapid titration of clozapine is an established risk factor for myocarditis. Some concomitant drugs or psychiatric or medical conditions can moderate myocarditis risk as well. Please rate with agree, not agree or no answer if you agree with the following statements:
Regarding valproate co-medication and increased myocarditis risk --> Do not use if clinically justifiable, or consider slower titration speed and enhanced monitoring.

**Q7b**

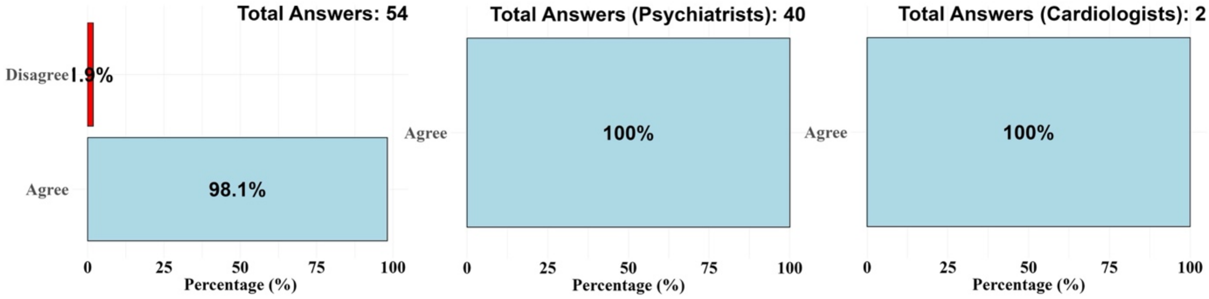


Not only a more rapid titration of clozapine is an established risk factor for myocarditis. Some concomitant drugs or psychiatric medical conditions can moderate myocarditis risk as well. Please rate with agree, not agree or no answer if you agree with the following statements:
Regarding CYP1A2-inhibitors from somatic medicine and possibly increased myocarditis risk (estrogen-containing oral contraceptives) --> Consider slower titration speed and enhanced monitoring.

**Q7c**

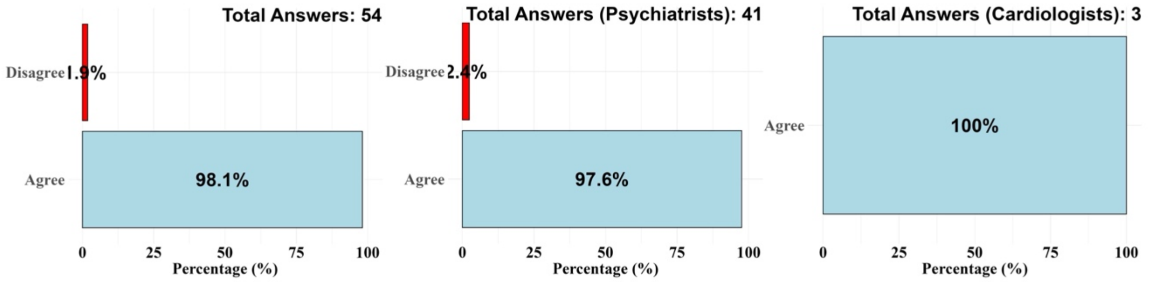


Not only a more rapid titration of clozapine is an established risk factor for myocarditis. Some concomitant drugs or psychiatric or medical conditions can moderate myocarditis risk as well. Please rate with agree, not agree or no answer if you agree with the following statements:
Regarding Asian ancestry or original inhabitants from the Americas and possibly increased myocarditis risk
--> Consider slower titration speed and enhanced monitoring.

**Q7d**

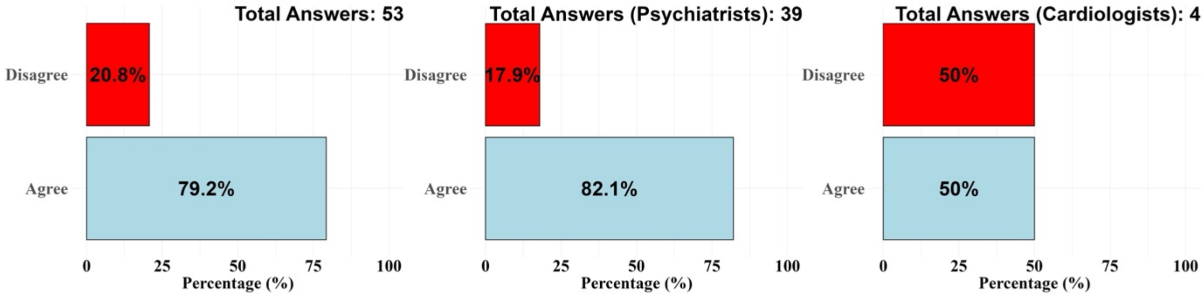


Not only a more rapid titration of clozapine is an established risk factor for myocarditis. Some concomitant drugs or psychiatric or medical conditions can moderate myocarditis risk as well. Please rate with agree, not agree or no answer if you agree with the following statements:
Regarding Obesity and increased myocarditis risk --> consider slower titration speed and enhanced monitoring.

**Q7e**

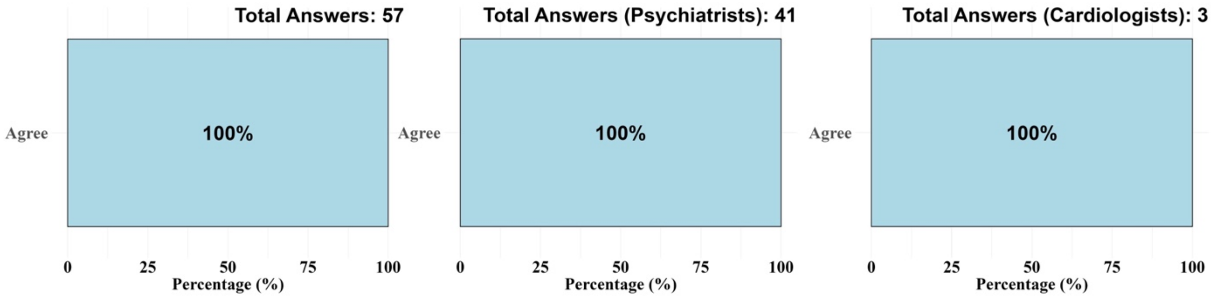


Not only a more rapid titration of clozapine is an established risk factor for myocarditis. Some concomitant drugs or psychiatric or medical conditions can moderate myocarditis risk as well. Please rate with agree, not agree or no answer if you agree with the following statements:
Regarding potent CYP1A2-inhibitors from psychiatry (e.g. fluvoxamine) and possibly increased myocarditis risk
--> Do not use if clinically justifiable, or consider slower titration speed and enhanced monitoring

**Q7f**

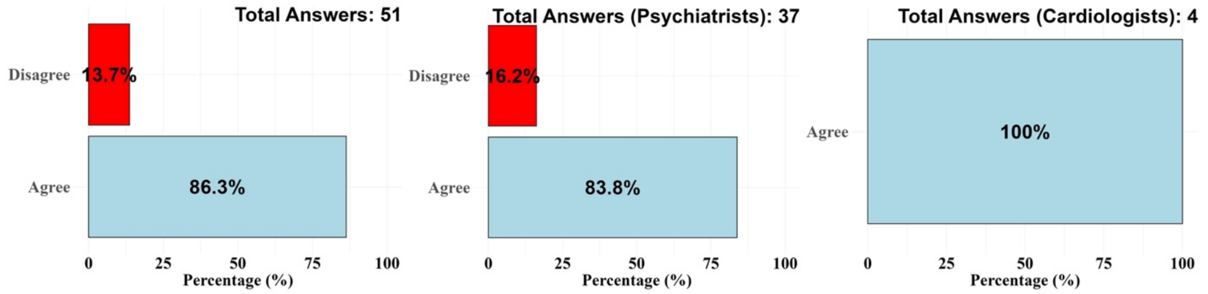


Not only a more rapid titration of clozapine is an established risk factor for myocarditis. Some concomitant drugs or psychiatric or medical conditions can moderate myocarditis risk as well. Please rate with agree, not agree or no answer if you agree with the following statements:
Regarding alcohol misuse and increased myocarditis risk --> Recommend minimal substance use, or initiate substance use treatment.

## Q7g

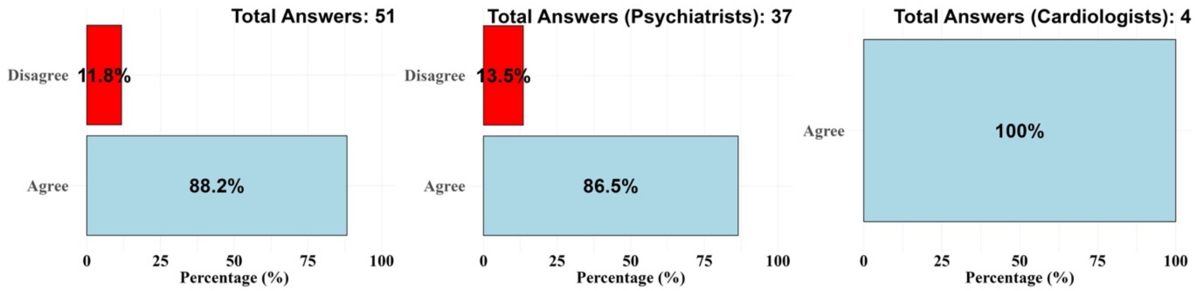


Not only a more rapid titration of clozapine is an established risk factor for myocarditis. Some concomitant drugs or psychiatric or medical conditions can moderate myocarditis risk as well. Please rate with agree, not agree or no answer if you agree with the following statements:
Regarding i.v. drug use (i.e. cocaine) and increased myocarditis risk --> Recommend minimal substance use, or initiate substance use treatment.

## Q7h

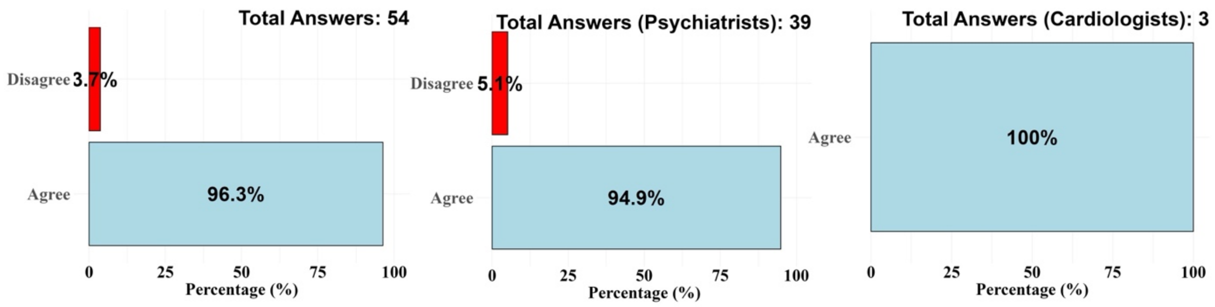


Not only a more rapid titration of clozapine is an established risk factor for myocarditis. Some concomitant drugs or psychiatric or medical conditions can moderate myocarditis risk as well. Please rate with agree, not agree or no answer if you agree with the following statements:
Regarding Chronic infectious diseases (i.e. HIV) and increased myocarditis risk --> Consider slower titration speed and enhanced monitoring.

**Q7i**

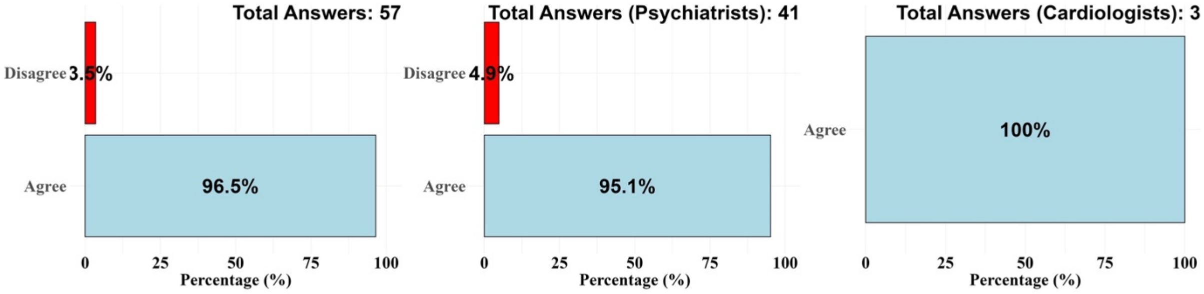


Not only a more rapid titration of clozapine is an established risk factor for myocarditis. Some concomitant drugs or psychiatric or medical conditions can moderate myocarditis risk as well. Please rate with agree, not agree or no answer if you agree with the following statements:
Regarding Concurrent bacterial/viral infection and increased myocarditis risk --> consider waiting periods if clinically justifiable and monitor CRP for return to baseline before clozapine initiation if possible, or consider slower titration speed and frequent monitoring.

**Q7j**

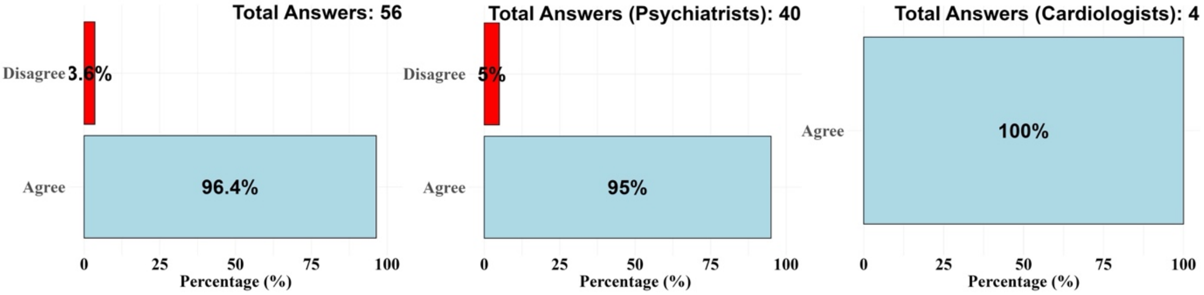


Not only a more rapid titration of clozapine is an established risk factor for myocarditis. Some concomitant drugs or psychiatric or medical conditions can moderate myocarditis risk as well. Please rate with agree, not agree or no answer if you agree with the following statements:
Regarding Pre-existing cardiac condition and overall increased vulnerability for cardiac adverse drug reactions
--> Interdisciplinary case discussion, slower titration speed and enhanced monitoring

**Q8**

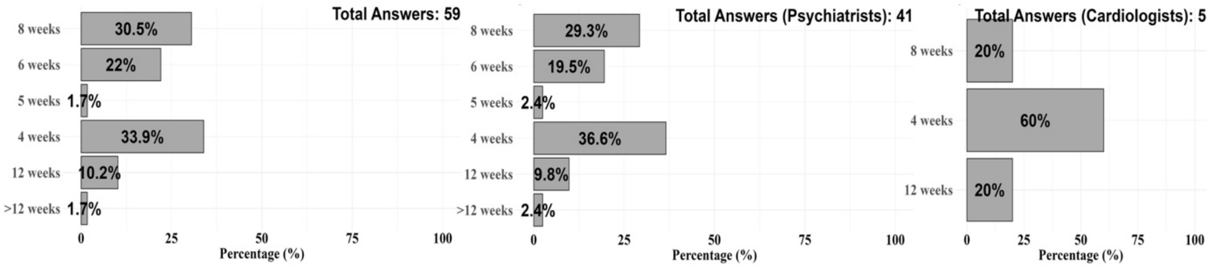


CAM following clozapine initiation outside of the first 4 weeks is rare, with research reporting that a vast majority of CAM develops between 18-21 days post initiation (Ronaldson et al., 2011). For how long should the minimum cardiac monitoring be continued in your opinion?

**Q9**

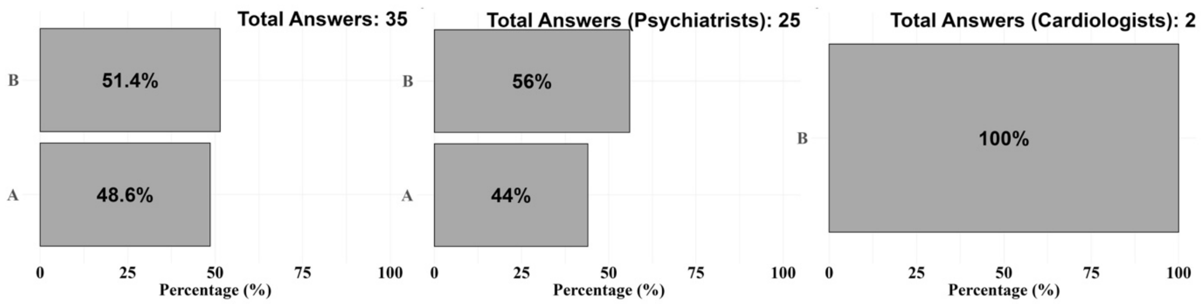


You suggested cardiac monitoring for longer than 4 weeks in the previous question. Which minimum scheme would you recommend for the period you defined? Please choose one option.

**A**

| Parameter | Minimum |
|---|---|
| CRP | weekly |
| FBC[1] | weekly |
| Hs cardiac troponin | weekly |
| NT-proBNP[6] | - |
| ECG | every 2 weeks |
| Heart rate | twice weekly |
| Oxygen saturation | weekly |
| Postural BP | weekly |
| temperature | twice weekly |

**B**

| Parameter | Minimum |
|---|---|
| CRP | every 2 weeks |
| FBC[1] | every 2 weeks |
| Hs cardiac troponin | every 2 weeks |
| NT-proBNP[6] | - |
| ECG | every 4 weeks |
| Heart rate | weekly |
| Oxygen saturation | weekly |
| Postural BP | weekly |
| temperature | weekly |

[1] only for cardiac monitoring, irrespective of regular ANC monitoring which is not covered in this survey

Abbreviation: FBC = full blood count

## Q10

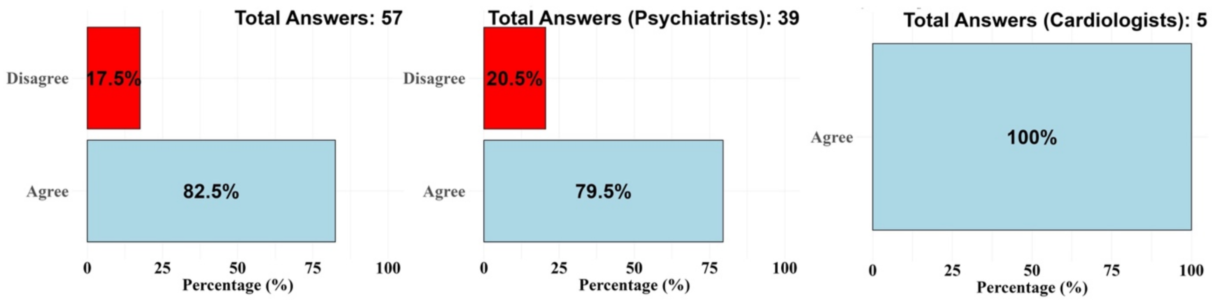


Would you agree with the following monitoring algorithm for clozapine initiation in weeks 1-4? The enhanced monitoring should be used in populations or situations as described above. Please rate with agree, not agree or no answer.

Algorithm for week 1-4: **CLOZAPINE INITIATION**

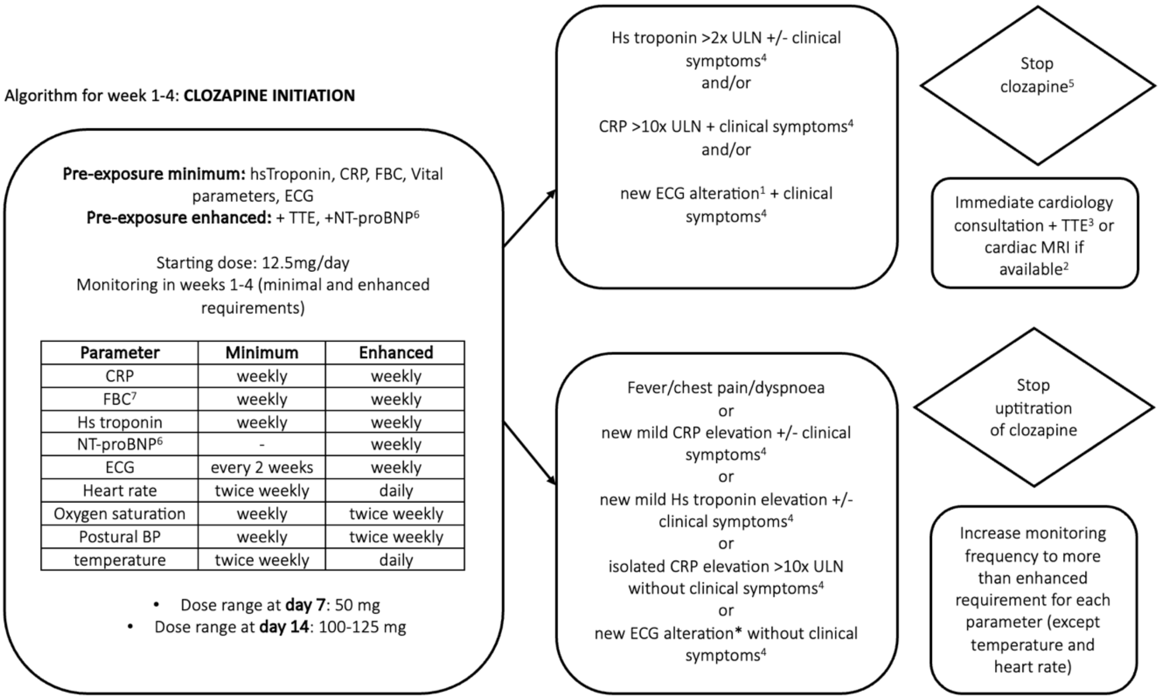


| Parameter | Minimum | Enhanced |
|---|---|---|
| CRP | weekly | weekly |
| FBC[7] | weekly | weekly |
| Hs troponin | weekly | weekly |
| NT-proBNP[6] | - | weekly |
| ECG | every 2 weeks | weekly |
| Heart rate | twice weekly | daily |
| Oxygen saturation | weekly | twice weekly |
| Postural BP | weekly | twice weekly |
| temperature | twice weekly | daily |

[1] i.e. new ST-segment and T-wave deviations
[2] Cardiac MRI shall be performed in cases when hsTroponin i/T > 99th percentile, NT-proBNP ≥ 125 pg/mL, or new significant rise from baseline beyond the biological and analytical variation of the assay used.
[3] Add NT-proBNP if cardiac imaging is not immediately available
[4] clinical symptoms highly suggestive of myocarditis in individuals treated with clozapine are: fever, chest pain or dyspnoea, and flu-like symptoms
[5] Potentially life-threatening conditions (such as torsades de points, cardiogenic shock, etc.) would trigger clozapine discontinuation before laboratory results are confirmed.
[6] if available in the respective jurisdiction
[7] only for cardiac monitoring, irrespective of regular ANC monitoring which is not covered in this survey

Abbreviation: CRP = c-reactive protein, FBC = full blood count

## Q11 - Rechallenge

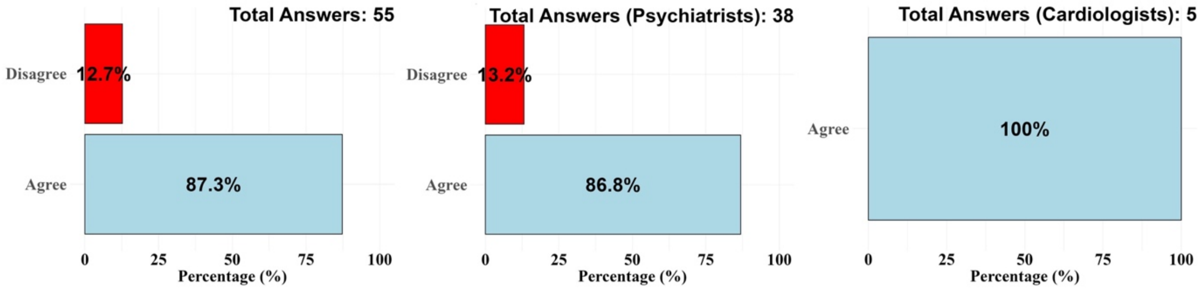


Would you agree with the following algorithm for clozapine rechallenge in cases of prior clozapine-associated myocarditis for weeks 1-4? The enhanced monitoring should be used in populations or situations as described above. Please rate with agree, not agree or no answer.

Algorithm for week 1-4: **CLOZAPINE RECHALLENGE**

**Pre-exposure minimum:** Hs (cardiac) troponin, CRP, FBC, Vital parameters, ECG, TTE
**Pre-exposure enhanced:** +NT-proBNP[6]

- LVEF must be at least 50%
- all cardiac lab results within range before re-challenge
- Cease Sodium Valproate
- Wait optimally more than 8 weeks after myocarditis resolution before clozapine re-challenge
- Always include TTE before re-challenge, cardiac MRI depending on case / clinical situation
- **STARTING DOSE: 6.25mg clozapine**
- Monitoring in weeks 1-4 (minimal and enhanced requirements)

| Parameter | Minimum | Enhanced |
|---|---|---|
| CRP | Twice Weekly | Twice weekly |
| FBC[7] | Weekly | Weekly |
| Hs troponin | Twice Weekly | Twice weekly |
| NT-proBNP[6] | - | Weekly |
| ECG | Weekly | Twice weekly |
| Heart rate | daily | daily |
| Oxygen saturation | Twice weekly | twice weekly |
| Postural BP | Twice weekly | twice weekly |
| temperature | daily | daily |

- Dose range at day 7: 25 mg
- Dose range at end of week 4: 100mg
- Uptitration more slowly than during myocarditis index episode

→ Clinical symptoms (Fever, chest pain, dyspnoea) and/or Any elevation of Hs troponin +/- clinical symptoms[4] and/or Severe elevation of CRP >10 x ULN

Stop clozapine[5]

Immediate cardiology consultation + TTE[3] or cardiac MRI if available[2]

→ Mild elevation of CRP without clinical symptoms[4] or New ECG alteration(s)[1] without clinical symptoms[4]

Stop uptitration of clozapine

Increase monitoring frequency to enhanced requirement for each parameter (or beyond, depending on parameter value)

[1] i.e. new ST-segment and T-wave deviations
[2] Cardiac MRI shall be performed in cases when hs Troponin i/T > 99th percentile, NT-proBNP ≥ 125 pg/mL or new significant rise from baseline beyond the biological and analytical variation of the assay used.
[3] Add NT-proBNP if cardiac imaging is not immediately available
[4] clinical symptoms highly suggestive of myocarditis in individuals treated with clozapine are: fever, chest pain or dyspnoea, and flu-like symptoms
[5] Potentially life-threatening conditions (such as torsades de points, cardiogenic shock etc.) would trigger discontinuation before laboratory results are confirmed.
[6] if available in the respective jurisdiction
[7] only for cardiac monitoring, irrespective of regular ANC monitoring which is not covered in this survey

Abbreviation: FBC = full blood count

## Q12 - Rechallenge

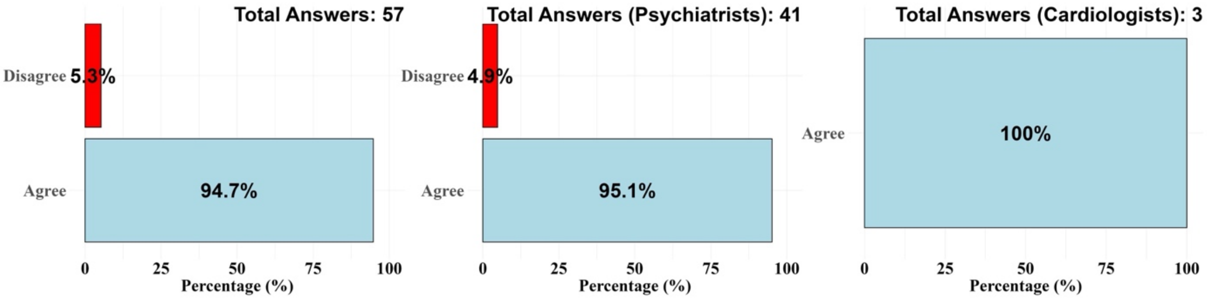


The majority recommended the following clozapine titration scheme for re-challenge. Please confirm with an agree, disagree, or no answer for the following majority-vote-agreed recommendation: 6.25mg starting dose on day 1.

## Q13 - Rechallenge

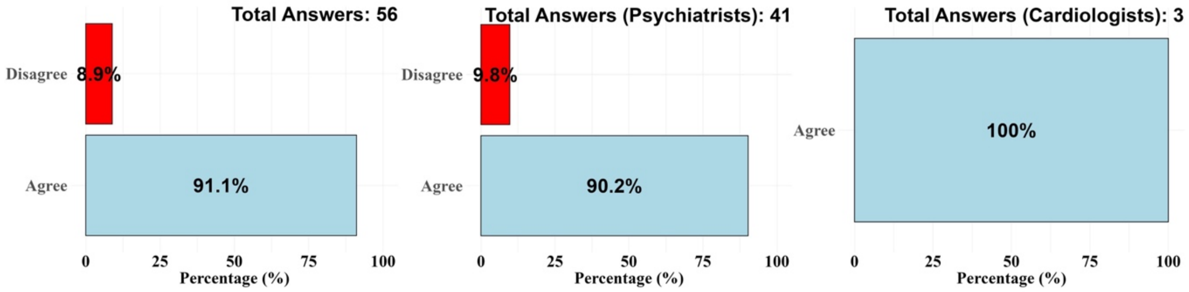


The majority recommended the following clozapine titration scheme for re-challenge. Please confirm with an agree, disagree, or no answer for the following majority-vote-agreed recommendation: 25mg at day 7.

## Q14 - Rechallenge

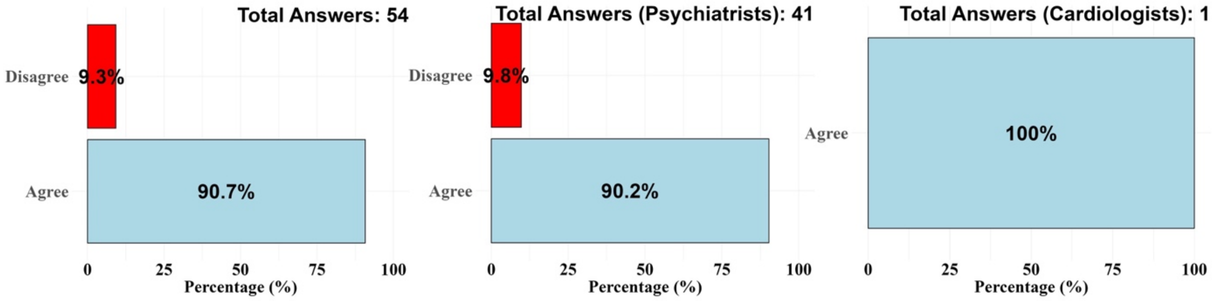


The majority recommended the following clozapine titration scheme for re-challenge. Please confirm with an agree, disagree, or no answer for the following majority-vote-agreed recommendation: 50mg at day 14.

## Q15 - Rechallenge

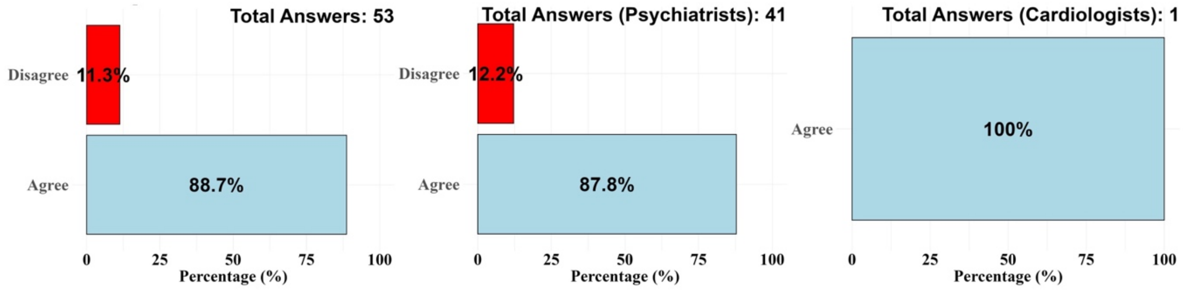


The majority recommended the following clozapine titration scheme for re-challenge. Please confirm with an agree, disagree, or no answer for the following majority-vote-agreed recommendation: 100mg at the end of week 4.

## Q16 - Rechallenge

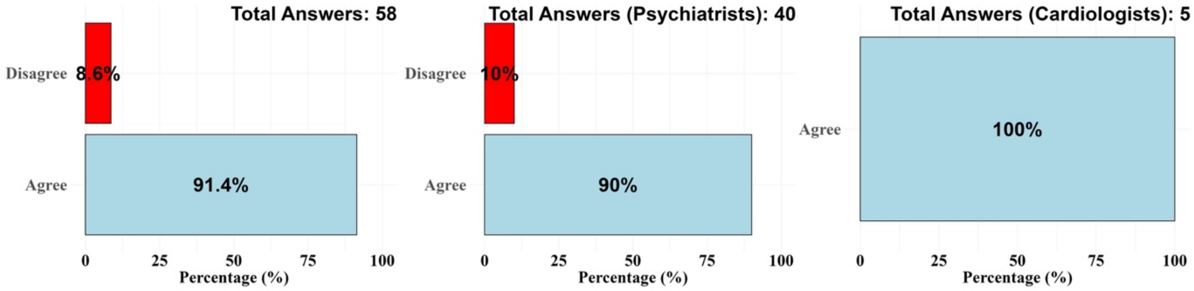


Mechanisms for recurrence of CAM during clozapine rechallenge are still not understood, but the risks for recurrence are accepted as being higher during clozapine rechallenge. In the first round, a majority agreed to a reduced monitoring protocol for weeks 5-8 in the absence of complications in weeks 1-4. Thus, would you agree with the following protocol for weeks 5-8, which steps down to weekly biomarker monitoring and twice weekly vital monitoring? Please rate with agree, not agree, no answer.

| Weeks 5-8 | | |
|---|---|---|
| **Parameter** | **Minimal** | **Enhanced** |
| CRP | Weekly | Twice weekly |
| FBC[2] | - | Weekly |
| hsTroponin | Weekly | Twice weekly |
| NT-proBNP[1] | - | Weekly |
| ECG | Every 2 weeks | Twice weekly |
| Heart rate | Twice weekly | daily |
| Oxygen saturation | weekly | twice weekly |
| Postural BP | weekly | twice weekly |
| temperature | Twice weekly | daily |

[1] not routinely available for monitoring in some jurisdictions
[2] only for cardiac monitoring, irrespective of regular ANC monitoring which is not covered in this survey
Abbreviation: FBC = full blood count
