# Supplementary material for: Multidisciplinary consensus on prevention, screening and monitoring of clozapine-associated myocarditis and clozapine rechallenge after myocarditis
Source: Br J Psychiatry. 2025 Jun 2;228(4):348–56. doi: 10.1192/bjp.2025.89 (PMC13051212; doi:10.1192/bjp.2025.89)
Supplement: Wagner et al. supplementary material 4 — Wagner et al. supplementary material [file S0007125025000893sup004.docx]

Affiliations of all authors from the CAM expert group are as follows:

**Psychiatrists:**

1. **Ofer Agid:** Centre for Addiction and Mental Health, Department of Psychiatry, University of Toronto, Canada
2. **Olatunde O. Ayinde:** Department of Psychiatry, College of Medicine, University of Ibadan, Ibadan, Nigeria.
3. **Andrea de Bartolomeis:** Clinical Section on Psychiatry and Psychology, Unit of Treatment Resistant Psychosis, Laboratory of Molecular and Translational Psychiatry, Department of Neuroscience and Staff Unesco Chair, University of Naples Federico II, Naples, Italy.
4. **Robert A. Bittner:** Department of Psychiatry, Psychosomatic Medicine and Psychotherapy, Goethe University Frankfurt, University Hospital, Frankfurt, Germany, Ernst Strüngmann Institute for Neuroscience (ESI) in Cooperation with Max Planck Society, Frankfurt, Germany.
5. **Chad A. Bousman:** Department of Medical Genetics, University of Calgary, Calgary, AB, Canada.
6. **Robert W. Buchanan:** Maryland Psychiatric Research Center, University of Maryland School of Medicine, Baltimore, Maryland, USA
7. **James MacCabe:** Department of Psychosis Studies, Institute of Psychiatry Psychology and Neuroscience, King's College London, London, UK.
8. **David Castle:** Department of Psychiatry, University of Tasmania, Sandy Bay, TAS, Australia, Centre for Mental Health Service Innovation, Department of Health, Tasmania, Australia.
9. **Sherry K. W. Chan:** Department of Psychiatry, School of Clinical Medicine, LKS Faculty of Medicine, The University of Hong Kong, Hong Kong Special Administrative Region, China; State Key Laboratory of Brain and Cognitive Sciences, The University of Hong Kong, Hong Kong Special Administrative Region, China.
10. **Leslie Citrome:** New York Medical College, Valhalla, NY, USA
11. **Scott R. Clark:** Discipline of Psychiatry, Adelaide University, Adelaide, SA, Australia.
12. **Dan Cohen:** Mental Health Services Noord-Holland-Noord, Alkmaar, the Netherlands; Dutch Clozapine Collaboration Group, Castricum, the Netherlands
13. **Christoph U. Correll:** Charité Universitätsmedizin, Augustenburger Platz 1, 13353 Berlin, Germany; The Zucker Hillside Hospital, 75-59 263rd Street, Glen Oaks, New York 11004, USA
14. **Robert O. Cotes:** Department of Psychiatry and Behavioral Sciences, Emory University School of Medicine, Atlanta, GA, USA.
15. **Robert McCutcheon:** Department of Psychiatry, University of Oxford, Oxford, OX3 7JX, England.
16. **Michael Davidson:** Department of Basic and Clinical Sciences, Psychiatry University of Nicosia Medical School, 2414, Nicosia, Cyprus; Minerva Neurosciences USA.
17. **Sonia Dollfus:** Caen Normandie University, PhIND, Inserm UMR S 1237, GIP Cyceron, Caen, 14000, France
18. **Brian O’Donoghue:** Department of Psychiatry, University College Dublin, Ireland; St Vincent's University Hospital, Elm Park, Dublin, Ireland; Centre for Youth Mental Health, University of Melbourne, VIC, Australia; Orygen, 35 Poplar Rd, Parkville, VIC 3052, Australia; Department of Psychiatry, Royal College of Surgeons, Ireland
19. **Serdar M. Dursun:** Department of Psychiatry, University of Alberta, Edmonton, Alberta, Canada
20. **Bjørn H. Ebdrup:**  Center for Neuropsychiatric Schizophrenia Research (CNSR), Mental Health Center, Glostrup, Copenhagen University Hospital – Mental Health Services CPH, Copenhagen, Denmark; Department of Clinical Medicine, Faculty of Health and Medical Sciences, University of Copenhagen, Copenhagen, Denmark
21. **Helio Elkis:** Department and Institute of Psychiatry- University of São Paulo Medical School- São Paulo, Brazil
22. **Susanna Every-Palmer:** Department of Psychological Medicine, University of Otago Wellington, Wellington, New Zealand.
23. **Peter Falkai:** Department of Psychiatry and Psychotherapy, LMU University Hospital, Nussbaumstrasse 7, 80336 Munich, Germany
24. **Emilio Fernandez-Egea:** Cambridge Psychosis Centre, Cambridgeshire and Peterborough NHS Foundation Trust and Department of Psychiatry, University of Cambridge, UK.
25. **Oliver Freudenreich:** Department of Psychiatry, Massachusetts General Hospital, Boston, Massachusetts, 02114, USA
26. **Ary Gadelha:** Department of Psychiatry, Escola Paulista de Medicina, Universidade Federal de São Paulo, São Paulo-SP, Brazil
27. **Wagner Farid Gattaz:** Laboratorio de Neurociencias (LIM 27), Instituto de Psiquiatria, Hospital das Clinicas HCFMUSP, Faculdade de Medicina, Universidade de Sao Paulo, Sao Paulo, SP, Brazil, Instituto Nacional de Biomarcadores em Neuropsiquiatria (INBION), Conselho Nacional de Desenvolvimento Científico e Tecnológico, São Paulo, Brazil.
28. **Fiona Gaughran:** Department of Psychosis Studies, Institute of Psychiatry, Psychology and Neuroscience, King's College London, London, UK; South London and Maudsley NHS Foundation Trust, London, UK.
29. **Sandeep Grover:** Department of Psychiatry, Postgraduate Institute of Medical Education and Research, Chandigarh, India.
30. **Ta-wei Guu:** Department of Old Age Psychiatry, Institute of Psychiatry Psychology and Neuroscience, King's College London, London, UK; Division of Psychiatry, Department of Internal Medicine, China Medical University Beigang Hospital, Yunlin, Taiwan.
31. **Jaime E. C. Hallak:** Neuroscience and Behavioural Sciences Department, Ribeirao Preto Medical School, University of Sao Paulo, Brazil
32. **Alkomiet Hasan:** Department of Psychiatry, Psychosomatics and Psychotherapy, University of Augsburg, Augsburg, Germany; DZPG (German Center for Mental Health), partner site München/Augsburg, Augsburg, Germany.
33. **Philipp Homan:** Department of Adult Psychiatry and Psychotherapy, University of Zurich, Lenggstrasse 31, 8032 Zurich, Switzerland
34. **William G. Honer:** Department of Psychiatry, University of British Columbia, Vancouver, Canada, and British Columbia Mental Health and Substance Use Services Research Institute, Vancouver, Canada
35. **Marte Z. van der Horst:** Department of Psychiatry, UMC Utrecht Brain Center, University Medical Center Utrecht, Utrecht University, Utrecht, Netherlands; GGNet Mental Health, Warnsveld, Netherlands.
36. **Oliver D. Howes:** King’s College London, IoPPN, 16 De Crespigny Park, London, UK; Imperial College London, Hammersmith Hospital, Du Cane Road, London, W12 0NN, UK
37. **Stefan Kaiser:** Department of Psychiatry, Geneva University Hospitals, Geneva, Switzerland
38. **Deanna L. Kelly:** Maryland Psychiatric Research Center, Department of Psychiatry, University of Maryland School of Medicine, Baltimore.
39. **Nicole Korman:** Addiction and Mental Health Services, Metro South Health Services, Australia; School of Medicine, University of Queensland, Brisbane, Australia.
40. **Vijay Kumar:** The Schizophrenia Clinic, Department of Psychiatry & Translational Psychiatry Laboratory, Neurobiology Research Centre, National Institute of Mental Health and Neurosciences, Bangalore, India
41. **Julia Lappin:** School of Psychiatry and Mental Health, University of New South Wales, Sydney, New South Wales, Australia.
42. **Victor Lasebikan:** Department of Psychiatry, College of Medicine, University of Ibadan, Ibadan. Department of Psychiatry, University College Hospital, Ibadan, Nigeria.
43. **Jimmy Lee:** North Region, Institute of Mental Health, Singapore and Lee Kong Chian School of Medicine, Nanyang Technological University, Singapore
44. **Jurjen J. Luykx:** GGZ inGeest Mental Health Care, Amsterdam, The Netherlands, Department of Psychiatry, Amsterdam Neuroscience and Amsterdam Public Health, Amsterdam UMC, Vrije Universiteit Amsterdam, Amsterdam, The Netherlands. Department of Psychiatry and Neuropsychology, School for Mental Health and Neuroscience, Maastricht University Medical Centre, Maastricht, The Netherlands.
45. **Victor Makanjuola:** Department of Psychiatry, University College Hospital, Ibadan, PMB 5116, Nigeria.
46. **Ahmed Malla:** Department of Psychiatry, Fattouma Bourguiba Hospital, Faculty of Medicine of Monastir, University of Monastir, Monastir, Tunisia.
47. **Tiago Reis Marques:** Department of Psychosis Studies, Institute of Psychiatry, Psychology and Neuroscience, King's College London, London, UK; Pasithea Therapeutics, Suite 500, 1111 Lincoln Road, Miami Beach, Florida 33139, USA
48. **Herbert Y. Meltzer:** Department of Psychiatry and Behavioral Sciences, Pharmacology and Neuroscience, Northwestern Feinberg School of Medicine, Chicago, IL.
49. **Fuminari Misawa:** Department of Neuropsychiatry, Yamanashi Prefectural Kita Hospital, Yamanashi, Japan.
50. **Shinichiro Nakajima:** Department of Neuropsychiatry, Keio University School of Medicine, Tokyo, Japan
51. **Jimmi Nielsen:** Unit for complicated schizophrenia, Mental Health Centre Glostrup, Copenhagen, Denmark; Copenhagen University Hospital, Copenhagen University, Copenhagen, Denmark
52. **Frederick C. Nucifora Jr.:** Department of Psychiatry and Behavioral Sciences, Johns Hopkins University School of Medicine, Baltimore, Maryland
53. **Akin Ojagbemi:** Department of Psychiatry University of Ibadan College of Medicine, Ibadan, Nigeria.
54. **Christos Pantelis:** Department of Psychiatry, The University of Melbourne & Melbourne Health; Parkville, VIC, Australia; Western Centre for Health Research & Education, University of Melbourne & Western Health, Sunshine Hospital, St Albans, Vic, Australia; Monash Institute of Pharmaceutical Sciences (MIPS), Monash University, Parkville, Vic, Australia
55. **Thomas J. Raedler:** Department of Psychiatry, Cumming School of Medicine, University of Calgary, Calgary, AB Canada
56. **Gary Remington:** Department of Psychiatry, University of Toronto, Centre for Addiction and Mental Health (CAMH), Toronto, Canada
57. **Jose M. Rubio:** Institute of Behavioral Sciences, Feinstein Institute for Medical Research, Northwell Health, New Hyde Park, NY 11042-1069, USA
58. **Surya Sandarsh:** Department of Psychiatry and Health Behavior, Medical College of Georgia, Augusta University, Augusta, GA, USA, East Central Regional Hospital, Augusta, GA, USA.
59. **Georgios Schoretsanitis:** The Zucker Hillside Hospital, Department of Psychiatry, Northwell Health, Glen Oaks, New York, Department of Psychiatry, Zucker School of Medicine at Northwell/Hofstra, Hempstead, Glen Oaks, New York, Department of Psychiatry, Psychotherapy and Psychosomatics, Hospital of Psychiatry, University of Zurich, Zurich, Switzerland.
60. **Peter F. J. Schulte:** Mental Health Service Noord-Holland-Noord, the Netherlands.
61. **Spyridon Siafis:** Technical University of Munich, Germany, TUM School of Medicine and Health, Department of Psychiatry and Psychotherapy, Germany; DZPG (German Center for Mental Health), partner site München/Augsburg, Augsburg, Germany.
62. **Dan Siskind:** Faculty of Medicine, University of Queensland, Brisbane, Australia and Addiction and Mental Health Service, Metro South Health, Brisbane, Australia
63. **Cynthia O. Siu:** Data Science, Toronto, Canada
64. **Marco Solmi:** Department of Psychiatry and Ottawa Hospital Research Institute Clinical Epidemiology Program, University of Ottawa, Ottawa, ON, Canada; Regional Centre for the Treatment of Eating Disorders and On Track: The Champlain First Episode Psychosis Program, Department of Mental Health, The Ottawa Hospital, Ottawa, ON, Canada; Department of Child and Adolescent Psychiatry, Charité Universitätsmedizin, Berlin, Germany.
65. **Jaana Suvisaari:** Finnish Institute for Health and Welfare, Finland.
66. **Takefumi Suzuki:** University of Yamanashi Faculty of Medicine, Department of Neuropsychiatry, Yamanashi, Japan
67. **David Taylor:** Institute of Pharmaceutical Sciences, King’s College London, Franklin-Wilkins Building, 150 Stamford Street, London SE1 9NH, UK; Pharmacy department, South London and Maudsley NHS Foundation Trust, Denmark Hill, London SE5 8AZ, UK
68. **Hiroyoshi Takeuchi:** Department of Neuropsychiatry, Keio University School of Medicine, Tokyo, Japan
69. **Si Tianmei:** Peking University Sixth Hospital, Peking University Institute of Mental Health, NHC Key Laboratory of Mental Health (Peking University), National Clinical Research Center for Mental Disorders Peking University Sixth Hospital, Beijing 100191, China
70. **Jagadisha Thirthalli:** Department of Psychiatry, National Institute of Mental Health and Neurosciences, Hosur Road, Bangalore 560 029, India.
71. **Masaru Tsukahara:** Department of Psychiatry, Okayama Psychiatric Medical Center, Okayama, Japan.
72. **Alp Ucok:** Istanbul University, Istanbul Faculty of Medicine, Istanbul, Turkey
73. **Vanteemar S** **Sreeraj:** Department of Psychiatry, National Institute of Mental Health and Neuro Sciences, Bengaluru, Karnataka India.
74. **Hélène Verdoux:** Univ. Bordeaux, Inserm, Bordeaux Population Health Research Center, team pharmacoepidemiology, Bordeaux, France.
75. **Jentien M. Vermeulen:** Department of Psychiatry, Amsterdam University Medical Centers, University of Amsterdam, Amsterdam, the Netherlands.
76. **Ganesan Venkatasubramanian:** Department of Psychiatry, National Institute of Mental Health and Neurosciences, Bengaluru, India
77. **Elias Wagner:** Department of Psychiatry, Psychotherapy and Psychosomatics, Medical Faculty, University of Augsburg, BKH Augsburg, Augsburg, Germany; Evidence-based Psychiatry and Psychotherapy, Faculty of Medicine, University of Augsburg, Augsburg, Germany
78. **James T. R. Walters:** Centre for Neuropsychiatric Genetics and Genomics, Division of Psychological Medicine and Clinical Neurosciences, Cardiff University, Cardiff, Wales.
79. **Yu Xin:** Peking University Sixth Hospital, Peking University Institute of Mental Health, NHC Key Laboratory of Mental Health (Peking University), National Clinical Research Center for Mental Disorders (Peking University Sixth Hospital), Beijing, China.

**Cardiologists:**

1. **Abiodun M. Adeoye:** College of Medicine, University of Ibadan, Nigeria.
2. **Brian Barr:** Department of Medicine, University of Maryland School of Medicine, Baltimore, MD, USA.
3. **Mathew K. Burrage:** Faculty of Medicine, University of Queensland, Brisbane, Australia, and Department of Cardiology, Ipswich Hospital, Queensland, Australia.
4. **Alida L. P. Caforio:** Cardiology, Department of Cardiac Thoracic Vascular Sciences and Public Health, University of Padova, Padova, Italy.
5. **Richard F. Choi:** Division of Cardiology. St. Joseph's Health Centre, University of Toronto, Toronto, Canada
6. **Leslie T. Cooper:** Department of Cardiovascular Medicine, Mayo Clinic in Florida, 4500 San Pablo, Jacksonville, USA.
7. **Theresa A McDonagh:** Department of Cardiology King's College Hospital, London, UK.
8. **Kevin O‘Gallagher:** Cardiovascular Department, King's College Hospital NHS Foundation Trust London, London, UK, School of Cardiovascular and Metabolic Medicine & Sciences, British Heart Foundation Centre of Research Excellence, King's College London, London, UK.
9. **Mattia Galli:** Maria Cecilia Hospital, GVM Care & Research, Cotignola, Italy.
10. **Nisha A. Gilotra:** Division of Cardiology, Department of Medicine, Johns Hopkins University School of Medicine, Baltimore, MD, USA
11. **Ulrich Grabmaier:** Department of Medicine I, LMU University Hospital, LMU Munich, Munich, Germany.
12. **Heikki V. Huikuri:** Research Unit of Biomedicine and Internal Medicine, Medical Research Center Oulu, University of Oulu, Oulu University Hospital, Oulu, Finland.
13. **Florian Leuschner:** Department of Cardiology, Angiology and Pneumology, University Hospital Heidelberg, 69120 Heidelberg, Germany; German Centre for Cardiovascular Research (DZHK), Partner Site Heidelberg/Mannheim, 69120 Heidelberg, Germany. .
14. **Antonio de Marvao:** Department of Women and Children's Health, King's College London, UK, British Heart Foundation Centre of Research Excellence, School of Cardiovascular Medicine and Sciences, King's College London, UK, Medical Research Council, London Institute of Medical Sciences, Imperial College London, UK.
15. **Sophie I. Mavrogeni:** Onassis Cardiac Surgery Center, Kapodistrian University of Athens, Athens, Greece.
16. **Narbeh Melikian:** Cardiovascular Department, King's College Hospital NHS Foundation Trust London, UK, School of Cardiovascular and Metabolic Medicine & Sciences, British Heart Foundation Centre of Research Excellence, King's College London London, UK.
17. **Okechukwu S. Ogah:** Neuroscience and Ageing Research Unit, Institute for Advanced Medical Research and Training, College of Medicine, University of Ibadan, Ibadan, Nigeria, Department of Medicine, University College Hospital/College of Medicine, University of Ibadan, Ibadan, Nigeria.
18. **Philip Raake:** I. Medical Department, Cardiology, Pneumology, Endocrinology and Intensive Care Medicine, University Hospital Augsburg, University of Augsburg, Augsburg, Germany.
19. **Stuart D. Rosen:** Immuno-Oncology Clinical Network, UK, London Northwest University Healthcare NHS Trust, London, UK, Clatterbridge Cancer Centre NHS Foundation Trust, Wirral, UK.
20. **Carsten Tschöpe:** Department of Cardiology, Deutsches Herzzentrum der Charité (DHZC), Angiology and Intensive Medicine (Campus Virchow) and German Centre for Cardiovascular Research (DZHK)- partner site Berlin, Charité Universitätsmedizin Berlin, Berlin, Germany, Berlin Institute of Health (BIH) at Charité - Center for Regenerative Therapies, Universitätsmedizin Berlin, Berlin, Germany.

**Pharmacists:**

1. **Toine C. G. Egberts:** Division of Pharmacoepidemiology & Clinical Pharmacology, Utrecht Institute for Pharmaceutical Sciences, Utrecht, The Netherlands, Department of Clinical Pharmacy, University Medical Centre Utrecht, Utrecht, The Netherlands.
2. **Siobhan Gee:** Pharmacy Department, South London and Maudsley NHS Foundation Trust, London, UK, Faculty of Life Sciences and Medicine, King's College London, London, UK.
3. **Caroline Hynes:** Pharmacy Department, Saint John of God Hospital, Co Dublin, Ireland
4. **Glynis Ivin:** South London and Maudsley NHS Foundation Trust Pharmacy, London, UK.
5. **Wolfgang Kämmerer:** Pharmacy Department, University of Augsburg, Medical Faculty, D-86156, Augsburg, Germany.
6. **Alexandra Blackfield:** Pharmacy Department, Royal Hobart Hospital, Tasmania, Australia
7. **Cynthia Mach:** Pharmacy Department, Royal Hobart Hospital, Tasmania, Australia
8. **Orla Macdonald:** Oxford Health NHS Foundation Trust, Oxford OX3 7JX, UK.
9. **Katie Mellor:** Department of General Surgery, Betsi Cadwaladr University Health Board, Bangor, LL57 2PW, Wales, UK
10. **Boon Tat Ng:** Department of Pharmacy, Institute of Mental Health, Singapore.
11. **Reza Rafizadeh:** Department of Experimental Medicine, University of British Columbia, Vancouver, BC, Canada, Department of Psychiatry, University of British Columbia, Vancouver, BC, Canada, Faculty of Pharmaceutical Sciences, University of British Columbia, Vancouver, BC, Canada, BC Mental Health and Substance Use Services, Vancouver, BC, Canada, BC Psychosis Program, UBC Hospital, Vancouver, BC, Canada, Lower Mainland Pharmacy Services, Vancouver, BC, Canada.
12. **Canjun Ruan:** The National Clinical Research Centre for Mental Disorders, Beijing Key Laboratory of Mental Disorders, and Laboratory of Clinical Psychopharmacology, Beijing Anding Hospital, Capital Medical University, Beijing, China, Advanced Innovation Center for Human Brain Protection, Capital Medical University, Beijing, China.
13. **Stephen Saklad:** College of Pharmacy, Pharmacotherapy Division, The University of Texas at Austin, 7703 Floyd Curl Drive, MC 6220, San Antonio, TX, 78229-3900, USA, Long School of Medicine, Pharmacotherapy Education and Research Center, UT Health San Antonio, San Antonio, TX, 78229-3900, USA.
14. **Lesley Smith:** Pharmacy Department, Princess Alexandra Hospital, Brisbane, Australia.
15. **Timothy Tanzer:** Faculty of Medicine, University of Queensland, Brisbane, Australia; Pharmacy Department, Princess Alexandra Hospital, Brisbane, Australia; School of Pharmacy, The University of Queensland, Brisbane, Australia.
16. **Nga Tran:** St Vincent's Mental Health, Melbourne, VIC, Australia Department of Psychiatry, The University of Melbourne, Melbourne, VIC, Australia.
17. **Eromona Whiskey:** South London and Maudsley NHS Foundation Trust, London, UK, King's College London, London, UK.
18. **Karl Winckel:** University of Queensland, Brisbane.
19. **Yulan Xiong:** Department of Neuroscience, University of Connecticut School of Medicine, Farmington, CT, USA.

**Medical Pharmacologist:**

1. [**François R Girardin**](https://url.avanan.click/v2/r02/___https://pubmed.ncbi.nlm.nih.gov/?term=Girardin+FR&cauthor_id=38663923___.YXAxZTpjYW1icmlkZ2Vvcmc6YTpvOjIxYTQzZDI0ZWE5NTk0ZTU4NWQzM2NkNDljYTY3ZDVkOjc6ZWU3ZDpmYTFhYWFkZTRkNGE3OTgzODM1NTQ5NjNhYTcwM2MzMzJmOTkxNWExZDkyYjliZTNkNjQ5ZjhkM2FmZjE0NzNiOnA6VDpG)**:** Division of clinical Pharmacology, Department of Medicine, Centre Hospitalier Universitaire Vaudois (CHUV) and University of Lausanne, Lausanne, Switzerland.

**Nurses:**

1. **Valerie Powell:** Centre of Addiction and Mental Health (CAMH), Toronto, Canada
2. **Sharon Locke:** Addiction and Mental Health Services, Princess Alexandra Hospital, Metro South Hospital and Health Service, Australia

## Conflicts of Interest

The authors declare that there are no conflicts of interest in relation to the subject of this study. General declaration of potential conflict of interests: EW was invited to advisory boards from Recordati, Teva and Boehringer Ingelheim. CUC has been a consultant and/or advisor to or has received honoraria from: AbbVie, Acadia, Adock Ingram, Alkermes, Allergan, Angelini, Aristo, Biogen, Boehringer-Ingelheim, Bristol-Meyers Squibb, Cardio Diagnostics, Cerevel, CNX Therapeutics, Compass Pathways, Darnitsa, Delpor, Denovo, Eli Lilly, Gedeon Richter, Hikma, Holmusk, IntraCellular Therapies, Jamjoom Pharma, Janssen/J&J, Karuna, LB Pharma, Lundbeck, MedInCell, Merck, Mindpax, Mitsubishi Tanabe Pharma, Maplight, Mylan, Neumora Therapeutics, Neurocrine, Neurelis, Newron, Noven, Novo Nordisk, Otsuka, PPD Biotech, Recordati, Relmada, Reviva, Rovi, Sage, Saladax, Sanofi, Seqirus, SK Life Science, Sumitomo Pharma America, Sunovion, Sun Pharma, Supernus, Tabuk, Takeda, Teva, Tolmar, Vertex, Viatris and Xenon Pharmaceuticals. He provided expert testimony for Janssen, Lundbeck and Otsuka. He served on a Data Safety Monitoring Board for Compass Pathways, Denovo, IntraCellular Therapies, Lundbeck, Relmada, Reviva, Rovi, Supernus, and Teva. He has received grant support from Boehringer-Ingelheim, Janssen and Takeda. He received royalties from UpToDate and is also a stock option holder of Cardio Diagnostics, Kuleon Biosciences, LB Pharma, Medlink, Mindpax, and Quantic. ODH reports investigator-initiated research funding from and/or participation in advisory/ speaker meetings organised by Abbvie, Angellini, Autifony, Biogen, Boehringer-Ingelheim, Delix, Eli Lilly, Elysium, Heptares, Global Medical Education, Invicro, Jansenn, Karuna, Lilly, Lundbeck, Merck, Neumora, Neurocrine, Ontrack/ Pangea, Otsuka, Sunovion, Teva, Recordati, Roche, Rovi and Viatris/ Mylan. He was previously a part-time employee of Lundbeck A/v. DS is funded in part by an NHMRC EL2 Investigator Grant (GNT 1194635) and declares that as associate editor of the *British Journal of Psychiatry*, he did not take part in the review or decision-making process of the paper. MS received honoraria/has been a consultant for Angelini, AbbVie, Boehringer Ingelheim, Lundbeck, Otsuka. AH was a member of advisory boards of Boehringer Ingelheim, Lundbeck, Janssen, Otsuka, Rovi and Recordati and received paid speakership by these companies as well as by AbbVie and Advanz. He is the editor of the German schizophrenia guideline.

## Conflict of interest from CAM expert group:

**Psychiatrists:**

1. **Ofer Agid:** has been a consultant and/ or advisor to or has received honoraria as follows: Advisory Board/ Consultant: Janssen-Ortho (Johnson & Johnson); Otsuka; Lundbeck; Allergan/Abbvie; Speaker: Janssen-Ortho (Johnson & Johnson); Lundbeck, Otsuka, Mylan Pharmaceuticals; Research Contracts: Janssen-Ortho (Johnson & Johnson); Otsuka; Boehringer Ingelheim.
2. **Olatunde O. Ayinde:** has no conflict of interest to declare.
3. **Andrea de Bartolomeis:** has received research support from Janssen, Lundbeck, and Otsuka and lecture fees for unrestricted educational meeting from Chiesi, Lundbeck, Roche, Sunovion, Viatris, HIKMA, Tabuk, Recordati, Angelini, Gedeon Richter and Takeda; he has served on advisory boards for Eli Lilly, Jansen, Lundbeck, Otsuka, Roche, Takeda, Chiesi, Recordati, Angelini, Viatris, Newron, Gedeon Richter.
4. **Robert A. Bittner:** was a member of advisory boards of Newron and Boehringer Ingelheim and received paid speakership by Recordati and Boehringer Ingelheim
5. **Chad A. Bousman:** has no conflict of interest to declare.
6. **Robert W. Buchanan:** is a DSMB member: Merck, Newron, and Roche; Advisory Board: Acadia, Karuna, Merck, and Neurocrine
7. **James MacCabe:** has no conflict of interest to declare.
8. **David Castle:** has no conflict of interest to declare.
9. **Sherry K. W. Chan:** has no conflict of interest to declare.
10. **Leslie Citrome:** has served as a consultant to AbbVie/Allergan, Acadia, Adamas, Alkermes, Angelini, Astellas, Avanir, Axsome, Biogen, BioXcel, Boehringer Ingelheim, Cadent Therapeutics, Cerevel, Clinilabs, COMPASS, Delpor, Eisai, Enteris BioPharma, HLS Therapeutics, Idorsia, INmune Bio, Impel, Intra-Cellular Therapies, Janssen, Karuna, Lundbeck, Luye, Lyndra, MapLight, Marvin, Medavante-ProPhase, Merck, Mitsubishi-Tanabe Pharma, Neumora, Neurocrine, Neurelis, Noema, Novartis, Noven, Otsuka, Ovid, Praxis, Recordati, Relmada, Reviva, Sage, Sumitomo/Sunovion, Supernus, Teva, University of Arizona, Vanda,  Wells Fargo, and one-off ad hoc consulting for individuals/entities conducting marketing, commercial, or scientific scoping research; speaker for AbbVie/Allergan, Acadia, Alkermes, Angelini, Axsome, BioXcel, Eisai, Idorsia, Intra-Cellular Therapies, Janssen, Lundbeck, Neurocrine, Noven, Otsuka, Recordati, Sage, Sunovion, Takeda, Teva, and CME activities organized by medical education companies such as Medscape, NACCME, NEI, Vindico, and Universities and Professional Organizations/Societies; owns stocks (small number of shares of common stock): Bristol-Myers Squibb, Eli Lilly, J & J, Merck, Pfizer purchased > 10 years ago, stock options: Reviva; and has earned royalties/publishing income from Taylor & Francis (Editor-in-Chief, Current Medical Research and Opinion, 2022-date), Wiley (Editor-in-Chief, International Journal of Clinical Practice, through end 2019), UpToDate (reviewer), Springer Healthcare (book), Elsevier (Topic Editor, Psychiatry, Clinical Therapeutics).
11. **Scott R. Clark:** has participated in advisory and educational boards and received speaker’s fees from Janssen-Cilag, Lundbeck, Otsuka, and Servier; research funding from Janssen-Cilag, Lundbeck, Otsuka and Gilead; and data sharing from Viatris Australia.
12. **Dan Cohen:** has no conflict of interest to declare.
13. **Christoph U. Correll:** has been a consultant and/or advisor to or has received honoraria from: AbbVie, Acadia, Adock Ingram, Alkermes, Allergan, Angelini, Aristo, Biogen, Boehringer-Ingelheim, Bristol-Meyers Squibb, Cardio Diagnostics, Cerevel, CNX Therapeutics, Compass Pathways, Darnitsa, Delpor, Denovo, Eli Lilly, Gedeon Richter, Hikma, Holmusk, IntraCellular Therapies, Jamjoom Pharma, Janssen/J&J, Karuna, LB Pharma, Lundbeck, MedInCell, Merck, Mindpax, Mitsubishi Tanabe Pharma, Maplight, Mylan, Neumora Therapeutics, Neurocrine, Neurelis, Newron, Noven, Novo Nordisk, Otsuka, PPD Biotech, Recordati, Relmada, Reviva, Rovi, Sage, Saladax, Sanofi, Seqirus, SK Life Science, Sumitomo Pharma America, Sunovion, Sun Pharma, Supernus, Tabuk, Takeda, Teva, Tolmar, Vertex, Viatris and Xenon Pharmaceuticals. He provided expert testimony for Janssen, Lundbeck and Otsuka. He served on a Data Safety Monitoring Board for Compass Pathways, Denovo, IntraCellular Therapies, Lundbeck, Relmada, Reviva, Rovi, Supernus, and Teva. He has received grant support from Boehringer-Ingelheim, Janssen and Takeda. He received royalties from UpToDate and is also a stock option holder of Cardio Diagnostics, Kuleon Biosciences, LB Pharma, Medlink, Mindpax, and Quantic.
14. **Robert O. Cotes:** has no conflict of interest to declare.
15. **Robert A. McCutcheon:** has received speaker/consultancy fees from Karuna, Janssen, Boehringer Ingelheim, and Otsuka, and co-directs a company that designs digital resources to support treatment of mental illness.
16. **Michael Davidson:** is an employee of Minerva Neurosciences.
17. **Sonia Dollfus:** received honoraria for conferences from Otsuka, Janssen and Lundbeck
18. **Brian O’Donoghue:** has no conflict of interest to declare.
19. **Serdar M. Dursun:** has no conflicts of interest related to this article to declare.
20. **Bjørn H. Ebdrup:** is part of the Advisory Board of Boehringer Ingelheim, Lundbeck Pharma A/S; and has received lecture fees from Boehringer Ingelheim, Otsuka Pharma Scandinavia AB, and Lundbeck Pharma A/S.
21. **Helio Elkis:** received a research grant from FAPESP (São Paulo’s Research Support Foundation) and honoraria for participation as a member of advisory boards, speaker, or travel support from the following pharmaceutical companies: Aché, ADIUM, Boeheringer- Ingelheim Cristália, Daiichi- Sankyo, Janssen, Mantecorp- Hypera and Teva. .
22. **Susanna Every-Palmer:** has no conflict of interest to declare.
23. **Peter Falkai:** has no conflicts of interest to declare.
24. **Emilio Fernandez-Egea:** has received consultancy honoraria from Boehringer-Ingelheim (2022), Atheneum (2022) and Rovi (2022-23), speaker fees by Adamed (2022-24), Otsuka (2023) and Viatris (2024) and training and research material from Merz (2020). He *Is deputy editor of the* BJPsych and did not take part in the reviewer decision-making process of this paper
25. **Oliver Freudenreich:** Janssen, Karuna, Otsuka: research grant. Janssen, Karuna, Neurocrine, Vida: consultant honoraria.  UpToDate: royalties
26. **Ary Gadelha:** received a research grant from FAPESP (São Paulo's Research Support Foundation and honoraria for participation as a member of advisory boards, speaker, or travel support from the following pharmaceutical companies: Aché, Cristália, Daiichi- Sankyo, Janssen, Mantecorp- Hypera, Lundbeck, Teva and Boehringer- Ingelheim.
27. **Wagner Farid Gattaz:** has no conflict of interest to declare.
28. **Fiona Gaughran:** In the last three years, FG has received honoraria for talks from Boehringer Ingelheim, Lundbeck, Otsuka, Recordati.
29. **Sandeep Grover:** has no conflict of interest to declare.
30. **Ta-wei Guu:** has no conflict of interest to declare.
31. **Jaime E. C. Hallak:** has no conflict of interest related to this article to declare.
32. **Alkomiet Hasan:** is editor of the German schizophrenia guideline. He has received paid speakerships from Boerhringer-Ingelheim, Janssen, Otsuka, Rovi, Recordati, Advanz and AbbVie. He was a member of Boerhringer-Ingelheim, Rovi, Recordati, Otsuka, Lundbeck, and Janssen advisory boards.
33. **Philipp Homan:** has received grants and honoraria from Novartis, Lundbeck, Mepha, Janssen, OM Pharma, Boehringer Ingelheim, and Neurolite outside of this work.
34. **Marte Z. van der Horst:** has no conflict of interest to declare.
35. **William G. Honer:** served as a consultant to AbbVie, Translational Life Sciences, Newron and Boehringer Ingelheim.
36. **Oliver D. Howes:** reports investigator-initiated research funding from and/or participation in advisory/ speaker meetings organised by Abbvie, Angellini, Autifony, Biogen, Boehringer-Ingelheim, Delix, Eli Lilly, Elysium, Heptares, Global Medical Education, Invicro, Jansenn, Karuna, Lilly, Lundbeck, Merck, Neumora, Neurocrine, Ontrack/ Pangea, Otsuka, Sunovion, Teva, Recordati, Roche, Rovi and Viatris/ Mylan. He was previously a part-time employee of Lundbeck A/v.
37. **Stefan Kaiser:** has received advisory board honoraria from Boehringer Ingelheim and Exeltis.
38. **Deanna L. Kelly:** has no conflict of interest to declare.
39. **Nicole Korman:** has no conflict of interest to declare.
40. **Vijay Kumar:** has no conflict of interest to declare.
41. **Julia Lappin:** has no conflict of interest to declare.
42. **Victor Lasebikan:** No conflicts of interest.
43. **Jimmy Lee:** had received honoraria from or served as a consultant or advisory board member on Otsuka, Janssen, Lundbeck, Sumitomo Pharmaceuticals, Boehringer Ingelheim and ThoughtFull World Pte. Ltd.
44. **Jurjen J. Luykx:** has no conflict of interest to declare.
45. **Victor Makanjuola:** has no conflict of interest to declare.
46. **Ahmed Malla:** has no conflict of interest to declare.
47. **Tiago Reis Marques:** is an employee and shareholder of Pasithea Therapeutics, and he has received employee stock options. He has received advisory board or consultant fees from Viatris and Pfizer within the past three years.
48. **Herbert Y. Meltzer:** has no conflict of interest to declare.
49. **Fuminari Misawa:** has no conflict of interest to declare.
50. **Shinichiro Nakajima:** has received grants from Japan Society for the Promotion of Science (18H02755, 22H03002), Japan Agency for Medical Research and development (AMED), Japan Research Foundation for Clinical Pharmacology, Naito Foundation, Takeda Science Foundation, Uehara Memorial Foundation, Watanabe Foundation, and Osake-no-Kagaku Foundation within the past three years. SN has received an investigator-initiated clinical study grant from Asahi Quality & Innovations, Ltd. SN has received research support, manuscript fees or speaker’s honoraria from Sumitomo Pharma, Meiji- Seika Pharma, Otsuka Pharmaceutical, and MSD within the past three years.
51. **Jimmi Nielsen:** has no conflicts of interest do declare.
52. **Frederick C. Nucifora:** served on an advisory board for Newron.
53. **Akin Ojagbemi:** reports funding by the DHSC, NIHR and Wellcome Trust Global Health Research Partnership International Intermediate Fellowship (Ref: 220684/Z/20/Z).
54. **Christos Pantelis:** (in the last 3 years) received honoraria for talks at educational meetings and has served on an advisory boards for Lundbeck, Australia Pty Ltd, and TEVA Australia. C Pantelis was supported by a National Health and Medical Research Council (NHMRC) L3 Investigator Grant (1196508) and NHMRC Program Grant (ID: 1150083).
55. **Thomas J. Raedler:** has received honoraria from Abbvie, Boehringer Ingelheim, HLS, Lundbeck, Newron, Otsuka, Sunovion and Teva. He has participated in clinical trials funded by Biohaven, Boehringer Ingelheim, Otsuka, SyneuRx International and Teva.
56. **Gary Remington:** receives research support from the Canadian Institutes of Health Research (CIHR), University of Toronto, and HLS Therapeutics.
57. **Jose M. Rubio:** reports honoraria from Lundbeck, TEVA, Janssen, Karuna, research funds from Alkermes, Neurocrine, Saladax, and royalties from UpToDate.
58. **Surya Sandarsh:** has no conflict of interest to declare.
59. **Georgios Schoretsanitis:** has no conflict of interest to declare.
60. **Peter Raphael F. J. Schulte:** has no conflict of interest to declare.
61. **Spyridon Siafis:** has no conflicts of interest to declare.
62. **Dan Siskind:** is funded in part by an NHMRC EL2 Investigator Grant (GNT 1194635). As associate editor of the *British Journal of Psychiatry*, he did not take part in the review or decision-making process of the paper He has no other conflicts to declare.
63. **Cynthia O. Siu:** has no conflict of interest to declare.
64. **Marco Solmi:** received honoraria/has been a consultant for Angelini, AbbVie, Boehringer Ingelheim, Lundbeck, Otsuka.
65. **Jaana Suvisaari:** has no conflict of interest to declare.
66. **Takefumi Suzuki:** has received manuscript or speaker’s fees from Astellas, Eisai, Eli Lilly, Elsevier Japan, Janssen Pharmaceuticals, Kyowa Yakuhin, Lundbeck Japan, Meiji Seika Pharma, Mitsubishi Tanabe Pharma, MSD, Nihon Medi-Physics, Novartis, Otsuka Pharmaceutical, Shionogi, Shire, Sumitomo Pharma, Takeda Pharmaceutical, Tsumura, Viatris, Wiley Japan, and Yoshitomi Yakuhin, and research grants from Eisai, Mochida Pharmaceutical, Meiji Seika Pharma, Shionogi and Sumitomo Pharma.
67. **Hiroyoshi Takeuchi:** has received grants from Daiichi Sankyo, Novartis Pharma, and Otsuka; speaker fees from EA Pharma, Eisai, Janssen, Kyowa, Lundbeck, Meiji Seika Pharma, Mitsubishi Tanabe Pharma, MSD, Otsuka, Sumitomo Pharma, Takeda, and Yoshitomiyakuhin; and consulting fees from Boehringer Ingelheim, Bristol Myers Squibb, Janssen, Mitsubishi Tanabe Pharma, Ono, and Sumitomo Pharma.
68. **David Taylor:** has received research funding from Janssen, Otsuka; consultancy payments from Viatris, Merck and Idorsia; and holds shares in 428Pharma and Myogenes.
69. **Si Tianmei:** has received research support from Janssen, Lundbeck, Eli Lilly, Pfizer and Otsuka and lecture fees for unrestricted educational meeting from Janssen, Lundbeck, Sunovion, Otsuka, and Sumitomo Pharma; She has served on advisory boards for Jansen, Lundbeck, Otsuka.
70. **Jagadisha Thirthalli:** has no conflict of interest to declare.
71. **Masaru Tsukahara:** has no conflict of interest to declare.
72. **Alp Ucok:** has been a consultant and/or advisor to or has received honoraria as follows: Janssen, Abdi Ibrahim Otzuka, Nobel.
73. **Vanteemar S** **Sreeraj:** has no conflict of interest to declare
74. **Hélène Verdoux:** has no conflict of interest to declare.
75. **Jentien Vermeulen: has no conflicts of interest to declare.**
76. **Ganesan Venkatasubramanian:** has no conflicts of interest do declare.
77. **Elias Wagner:** has been invited to advisory boards from Boehringe Ingelheim, Teva and Recordati.
78. **James T. R. Walters:** has no conflict of interest to declare.
79. **Yu Xin:** has no conflicts to declare.

**Cardiologists:**

1. **Abiodun M. Adeoye:**
2. **Brian Barr:** has no conflict of interest to declare.
3. **Mathew K. Burrage:** is funded in part by a National Heart Foundation Postdoctoral Fellowship and has received honoraria from Astra Zeneca.
4. **Alida L. P. Caforio:** has no conflict of interest to declare.
5. **Richard F. Choi:** consultant and minor equity holder, HLS Therapeutics.
6. **Leslie T. Cooper:** consulting relationships with BMS, Cardiol Therapeutics, and Moderna. Equity Ownership in Stromal Therapeutics.
7. **Theresa A McDonagh:** has no conflict of interest to declare.
8. **Kevin O‘Gallagher:** has no conflict of interest to declare.
9. **Mattia Galli:** has no conflict of interest to declare.
10. **Nisha A. Gilotra: has been a consultant for Kiniksa Pharmaceuticals**
11. **Ulrich Grabmaier:** has no conflict of interest to declare.
12. **Heikki V. Huikuri:** has no conflict of interest to declare.
13. **Florian Leuschner:** has no conflict of interest to declare.
14. **Antonio de Marvao:** has no conflict of interest to declare.
15. **Sophie I. Mavrogeni:** has no conflict of interest to declare.
16. **Narbeh Melikian:** has no conflict of interest to declare.
17. **Okechukwu S. Ogah:** has no conflict of interest to declare.
18. **Philipp Raake:** has no conflict of interest to declare.
19. **Stuart D. Rosen:** has no conflict of interest to declare.
20. **Carsten Tschöpe:** has no conflict of interest to declare.

**Pharmacists:**

1. **Toine C. G. Egberts:** has no conflict of interest to declare.
2. **Siobhan Gee:** has no conflict of interest to declare.
3. **Caroline Hynes:** has no conflict of interest to declare.
4. **Glynis Ivin:** has no conflict of interest to declare.
5. **Wolfgang Kämmerer:** has no conflict of interest to declare.
6. **Alexandra Blackfield:** has no conflict of interest to declare.
7. **Cynthia Mach:** has no conflict of interest to declare.
8. **Orla Mcdonald:** has no conflict of interest to declare.
9. **Katie Mellor:** has no conflict of interest to declare.
10. **Boon Tat Ng:** has no conflict of interest to declare.
11. **Reza Rafizadeh:** has no conflict of interest to declare.
12. **Canjun Ruan:** has no conflict of interest to declare.
13. **Stephen Saklad:** has no conflict of interest to declare.
14. **Lesley Smith:** has no conflict of interest to declare.
15. **Timothy Tanzer:** has no conflict of interest to declare.
16. **Nga Tran:** has no conflict of interest to declare.
17. **Eromona Whiskey:** has no conflict of interest to declare.
18. **Karl Winckel:** has no conflict of interest to declare.
19. **Yulan Xiong:** has no conflict of interest to declare.

**Medical Pharmacologist:**

1. [**François R Girardin**](https://url.avanan.click/v2/r02/___https://pubmed.ncbi.nlm.nih.gov/?term=Girardin+FR&cauthor_id=38663923___.YXAxZTpjYW1icmlkZ2Vvcmc6YTpvOjIxYTQzZDI0ZWE5NTk0ZTU4NWQzM2NkNDljYTY3ZDVkOjc6ZWU3ZDpmYTFhYWFkZTRkNGE3OTgzODM1NTQ5NjNhYTcwM2MzMzJmOTkxNWExZDkyYjliZTNkNjQ5ZjhkM2FmZjE0NzNiOnA6VDpG)**:** has no conflict of interest to declare

**Nurses:**

1. **Valerie Powell:** has no conflict of interest to declare.
2. **Sharon Locke:** has no conflict of interest to declare.
